# FgPrp4 Kinase Is Important for Spliceosome B-Complex Activation and Splicing Efficiency in *Fusarium graminearum*

**DOI:** 10.1371/journal.pgen.1005973

**Published:** 2016-04-08

**Authors:** Xuli Gao, Qiaojun Jin, Cong Jiang, Yang Li, Chaohui Li, Huiquan Liu, Zhensheng Kang, Jin-Rong Xu

**Affiliations:** 1 State Key Laboratory of Crop Stress Biology for Arid Areas, College of Plant Protection, Northwest A&F University, Yangling, Shaanxi, China; 2 Dept. of Botany and Plant Pathology, Purdue University, West Lafayette, Indiana, United States of America; The University of North Carolina at Chapel Hill, UNITED STATES

## Abstract

*PRP4* encodes the only kinase among the spliceosome components. Although it is an essential gene in the fission yeast and other eukaryotic organisms, the *Fgprp4* mutant was viable in the wheat scab fungus *Fusarium graminearum*. Deletion of *FgPRP4* did not block intron splicing but affected intron splicing efficiency in over 60% of the *F*. *graminearum* genes. The *Fgprp4* mutant had severe growth defects and produced spontaneous suppressors that were recovered in growth rate. Suppressor mutations were identified in the *PRP6*, *PRP31*, *BRR2*, and *PRP8* orthologs in nine suppressor strains by sequencing analysis with candidate tri-snRNP component genes. The Q86K mutation in *FgMSL1* was identified by whole genome sequencing in suppressor mutant S3. Whereas two of the suppressor mutations in FgBrr2 and FgPrp8 were similar to those characterized in their orthologs in yeasts, suppressor mutations in Prp6 and Prp31 orthologs or *FgMSL1* have not been reported. Interestingly, four and two suppressor mutations identified in FgPrp6 and FgPrp31, respectively, all are near the conserved Prp4-phosphorylation sites, suggesting that these mutations may have similar effects with phosphorylation by Prp4 kinase. In FgPrp31, the non-sense mutation at R464 resulted in the truncation of the C-terminal 130 aa region that contains all the conserved Prp4-phosphorylation sites. Deletion analysis showed that the N-terminal 310-aa rich in SR residues plays a critical role in the localization and functions of FgPrp4. We also conducted phosphoproteomics analysis with FgPrp4 and identified S289 as the phosphorylation site that is essential for its functions. These results indicated that FgPrp4 is critical for splicing efficiency but not essential for intron splicing, and FgPrp4 may regulate pre-mRNA splicing by phosphorylation of other components of the tri-snRNP although itself may be activated by phosphorylation at S289.

## Author Summary

In eukaryotic organisms, many genes containing introns that need to be spliced by the spliceosome after transcription. Among all the spliceosome components, Prp4 is the only protein kinase. Unlike other organisms, deletion of the *FgPRP4* kinase gene was not lethal in the wheat scab fungus *Fusarium graminearum*. In this study, we found that *FgPRP4* is not essential for intron splicing but important for splicing efficiency. The *Fgprp4* mutant was not stable and produced spontaneous suppressors recovered in growth rate. Suppressor mutations were identified in the PRP6, PRP31, BRR2, and PRP8 orthologs, key components of the U4/U6-U5 complex in the spliceosome and *FgMSL1* by candidate gene or whole genome sequencing. We also showed that the N-terminal 310 amino acid region of FgPrp4 plays a critical role in its localization and functions of FgPrp4 and identified S289 as a critical phosphorylation site. Overall, our result indicated that FgPrp4 is important for splicing efficiency, possibly by phosphorylation of other spliceosome components.

## Introduction

Pre-mRNA splicing is mediated by the spliceosome that is formed by ordered interaction of the U1, U2, U4/U6, U5 snRNPs, and non-snRNP proteins [1]. U1 and U2 first interact with the 5’-splice site (5’-ss) and the branch point (BP) of the introns in pre-mRNA to generate the A complex. The A complex is then converted to the pre-catalytic B-complex by the integration of the preformed U4/U6-U5 tri-snRNP. Activation of the B-complex involves the unwinding of U4/U6 and dissociation of U1 and U4. Whereas the activated B-complex catalyzes the first step of splicing, the C complex catalyzes the second step of splicing to form mature mRNA [2].

Unwinding of U4/U6, a critical step during B-complex activation, is catalyzed by the Brr2 DExD/H-box family RNA helicase that recognizes the single-stranded region of U4 next to the Stem I of the U4/U6 [2]. The helicase activity of Brr2 is regulated by Prp8 and Snu114 to prevent premature unwinding of U4/U6 [1,2]. Prp6 and Prp31 also are two essential components of the U4/U6-U5 tri-snRNP. However, unlike Prp8 and Brr2, they lack structural domains with defined biochemical functions. Prp6 and Prp31 are associated with pre-catalytic spliceosomal complexes [3] but not with the activated- or post-catalytic spliceosomal complexes [4–7]. Prp6 interacts with the U4/U6 specific protein Prp31 and the U5 proteins Brr2 and Prp8 [8,9].

Many components of the spliceosome are conserved in eukaryotic organisms [10]. However, the budding yeast *Saccharomyces cerevisiae*, a model for studying spliceosome and intron splicing, lacks a distinct ortholog of Prp4, which is the only serine/threonine protein kinase among the spliceosome components [11]. In the fission yeast *Schizosaccharomyces pombe*, *prp4* is an essential gene required for intron splicing [11]. It phosphorylates the non-SR protein Prp1 and its kinase activity is essential for G1-S and G2-M transition in the cell cycle [12]. In humans, hPrp4 is specifically associated with the U4/U6 and U4/U6-U5 RNPs. It functionally interacts with hPrp6 (human ortholog of *S*. *pombe* Prp1), Prp31, Brr2, and Prp8, and plays an essential role in the catalytic activation of B-complex [3]. Phosphorylation of hPrp6 and hPrp31 by hPrp4 is required for stable integration of the tri-snRNP into the B-complex, and it has been characterized by phosphoproteomics analysis [11].

Whereas Prp4 is essential in *S*. *pombe*, deletion of its orthologous gene appears to be not lethal in *Fusarium graminearum* because the putative *Fgprp4* deletion mutant was identified in a systematic characterization study of its protein kinase genes. *F*. *graminearum* is the predominant species causing Fusarium head blight (FHB), one of the most important diseases on wheat and barley [13,14]. It causes severe yield losses and contaminates infested grains with harmful mycotoxins, including zearalenone and trichothecene mycotoxin deoxynivalenol (DON), a potent inhibitor of eukaryotic protein synthesis [15,16].

The *PRP4* orthologs are well conserved in filamentous fungi but none of them have been functionally characterized, including the model organisms *Neurospora crassa* and *Aspergillus nidulan*s. To our knowledge, *Fgprp4* is the only null mutant that is available for this well-conserved protein kinase gene among all the eukaryotic organisms. In this study, we further characterized the function of *FgPRP4* in intron splicing and suppressor mutations of the *Fgprp4* mutant. Our results showed that FgPrp4 is critical for splicing efficiency and FgPrp4 may regulate pre-mRNA splicing by phosphorylation of other tri-snRNP proteins. FgPrp4 itself may be phosphorylated at the N-terminal region by autophosphorylation or other protein kinases.

## Results

### *FgPRP4* is important for growth, differentiation, and pathogenesis

The Prp4 ortholog in *F*. *graminearum* (Fg04053) shares 57% identity with Prp4 of *S*. *pombe* but their homology is mainly in the kinase domain. Although it is conserved in other ascomycetes, a distinct Prp4 ortholog was absent in Saccharomycotina species except *Yarrowia lipolytica* (S1 Fig). Most of Saccharomycotina species, including *S*. *cerevisiae* and *Candida albicans*, may have lost the *PRP4* ortholog during evolution after massive intron loss [17].

**Fig 1:**
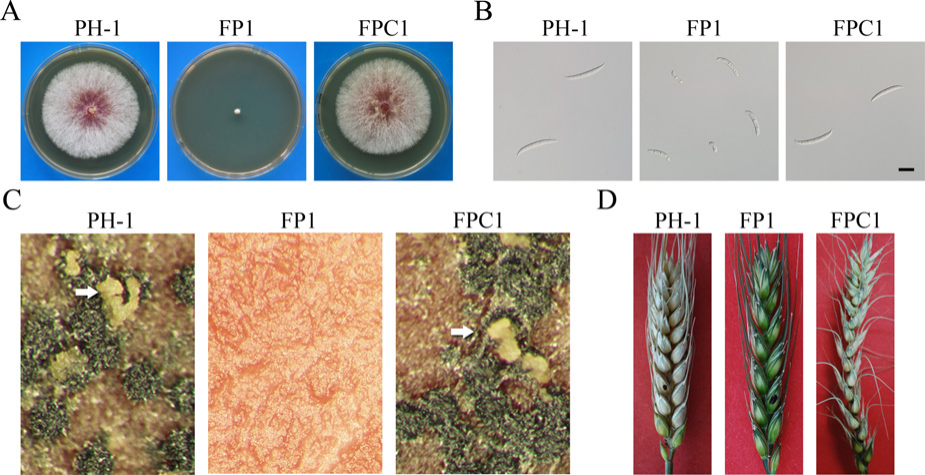
Defects of the *Fgprp4* mutant in growth, conidia morphology, sexual reproduction, and pathogenesis. (**A).** Three-day old PDA cultures of the wild type (PH-1), Δ*Fgprp4* mutant (FP1), and *Fgprp4/FgPRP4* complement strain (FPC1). (**B).** Conidia morphology of PH-1, FP1, and FPC1. Bar = 20 μm. **(C)**. Mating plates of PH-1, FP1, and FPC1. Ascospore cirrhi produced by black perithecia were marked with arrows. **(D).** Flowering wheat heads drop-inoculated with conidium suspensions of the same set of strains were photographed 14 days post-inoculation (dpi). The inoculated spikelets were marked with a black dot.

Unlike *prp4* in *S*. *pombe*, the putative *FgPrp4* mutant was viable in *F*. *graminearum* [18]. In this study we first confirmed the *Fgprp4* mutant by Southern blot analysis (S2 Fig). Careful examinations showed that the *Fgprp4* mutant had severe growth defects (Fig 1A) and rarely produced morphologically abnormal conidia (Fig 1B). The length of *Fgprp4* conidia (28.3 ± 7.1 μm) was approximately 45% shorter than that of wild-type conidia (51.2 ± 8.9 μm). Deletion of *FgPRP4* also reduced conidiation. Whereas the *Fgprp4* mutant produced 2.4 ± 1.7x10^4^ conidia/ml in 5-day-old CMC cultures, the wild type strain produced over 10^6^ conidia/ml under the same conditions. In addition, the *Fgprp4* mutant failed to produce perithecia on mating plates (Fig 1C) and was non-pathogenic in infection assays with flowering wheat heads (Fig 1D). To confirm its function, we re-introduced the full-length *FgPRP4* allele into the *Fgprp4* mutant strain FP1. The resulting *Fgprp4/FgPRP4* transformant FPC1 (Table 1) was similar to the wild type in growth rate, conidiation, sexual reproduction, and virulence (Fig 1). Therefore, deletion of *FgPRP4* is responsible for all the phenotypes observed in the *Fgprp4* mutant.

**Table 1:** Wild-type and transformants of *Fusarium graminearum* strains used in this study.

Strain	Brief description	References
PH-1	Wild-type	[56]
FP1	*Fgprp4* deletion mutant of PH-1	[18]
FPC1	*Fgprp4*/*FgPRP4* transformant of FP1	This study
FPN1	*Fgprp4*/*FgPRP4*-GFP transformant of FP1	This study
FPF1	*FgPRP4*-3xFLAG transformant of PH-1	This study
Suppressor strains S1-S49*	Spontaneous suppressor mutants of FP1	This study
S2Ma	*Fgprp4/ FgPRP31*^R464*^ mutant	This study
S17Ma	*Fgprp4/ FgPRP31*^L532P^ mutant	This study
FPN310	*Fgprp4*^ρ N1-310^-GFP transformant of FP1	This study
FPA2	*Fgprp4*^S289^A transformant of FP1	This study
FGSC17970	ectopic *stk*-57 (*prp4*) deletion transformant of *Neurospora crassa*	[33]

* 49 suppressor mutants derived from fast-growing sectors of the *Fgprp4* deletion mutant FP1.

### The N-terminal 310-aa SR-rich region is essential for FgPrp4 function and localization to the nucleus

**Fig 2:**
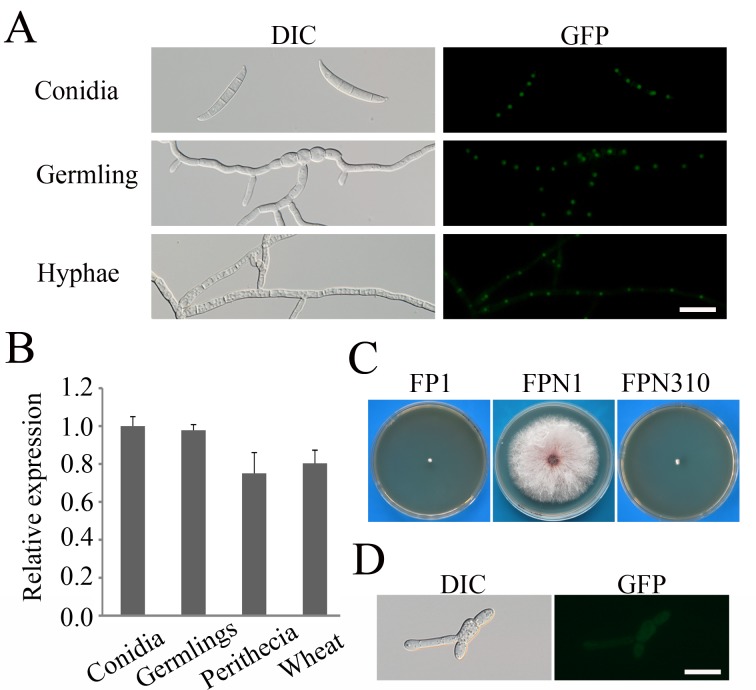
Assays for the function of the N-terminal 310 aa of FgPrp4. (**A).** Conidia, 12 h germlings, and hyphae of the *Fgprp4/FgPRP4-*GFP transformant (FPN1) were examined by DIC and epifluorescence microscopy. Bar = 20 μm. (**B).** The expression level of *FgPRP4* was assayed by qRT-PCR with RNA isolated from conidia, 12 h germlings, perithecia at 10 days post-fertilization, and infected wheat heads at 7 days post-inoculation (dpi). Mean and standard deviation were calculated with data from three independent biological replicates. The β-tubulin gene FGSG_06611 of *F*. *graminearum* was used as the internal control. (**C).** Three-day old PDA cultures of the *Fgprp4* mutant (FP1), *Fgprp4/FgPRP4*-GFP transformant (FPN1), and *Fgprp4/FgPRP4*^Δ1-310^-GFP transformant (FPN310). (**D).** 12 h germlings of transformant FPN310 were examined by DIC and epifluorescence microscopy. Bar = 20 μm.

To determine its subcellular localization, we fused GFP to the carboxyl-terminus of *FgPRP4* and transformed the *FgPRP4-*GFP construct into the *Fgprp4* mutant FP1. The resulting *FgPRP4*-GFP transformant FPN1 (Table 1) was normal in growth (S3 Fig), reproduction, and pathogenesis. When examined by epifluorescence microscopy, GFP signals of similar strength were observed in the nucleus in conidia, germlings, and hyphae (Fig 2A). When assayed by qRT-PCR, *FgPRP4* had similar expression levels in conidia, germlings, perithecia, and infected wheat heads (Fig 2B). These results indicate that *FgPRP4* is constitutively expressed in *F*. *graminearum* and its localization to the nucleus may be associated with its functions in the spliceosome.

Like hPrp4, FgPrp4 has a long N-terminal region that is rich in serine and arginine (SR-rich) and contains one putative nuclear localization signal (NLS). This N-terminal SR-rich domain of FgPrp4 is absent in its orthologs from *S*. *pombe* (S4 Fig). To determine its function, we generated the *FgPRP4*^ΔN310^-GFP construct deleted of the N-terminal 310 aa and transformed it into the *Fgprp4* mutant. The resulting *FgPRP4*^ΔN310^-GFP transformant had similar phenotypes with the original mutant (Fig 2C) and GFP signals in the cytoplasm (Fig 2D). These results indicate that the N-terminal region of FgPrp4 is essential for its localization and function in *F*. *graminearum*. Interestingly, *FgPRP4* has two isoforms based on our RNA-seq data (S5A Fig) [19]. Isoform A encodes the full-length FgPrp4 kinase as predicted by automated annotation. Isoform B has the retention of the forth intron and encodes a protein with the predicted C’-terminal 73 aa region replaced with 66 aa encoded by the retained intron (S5B Fig). The protein encoded by isoform B should have no kinase function because the protein kinase domain was disrupted (S5B). Nevertheless, isoform A accounted for over 85% of the *FgPRP4* transcripts in RNA-seq data of hyphae, conidia, and perithecia (S5A Fig). This observation was verified by qRT-PCR analysis (S5C Fig), indicating that isoform A is the predominant transcript of *FgPRP4*.

### Intron splicing efficiency is affected by deletion of *FgPRP4*

**Fig 3:**
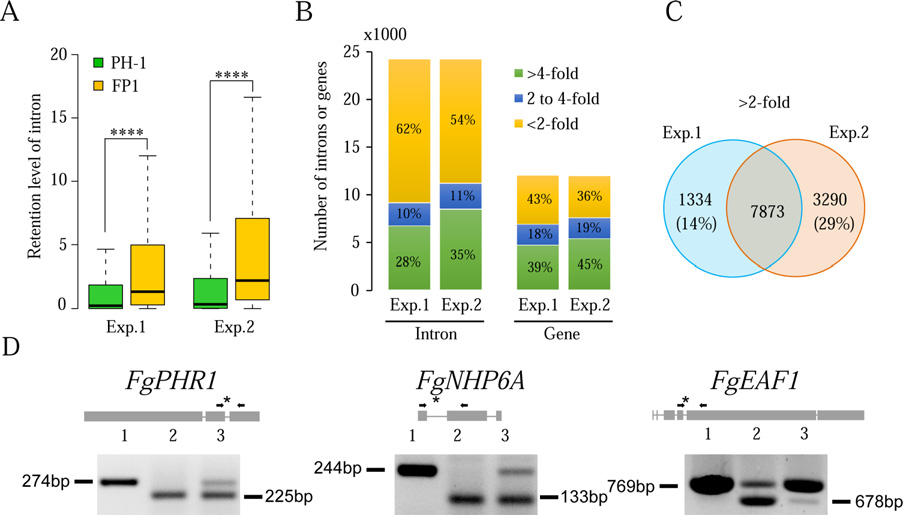
Effects of *FgPRP4* deletion on intron splicing. (**A).** Box-plot comparison of intron retention levels between the wild type (PH-1) and *Fgprp4* mutant (FP1) in replica experiments. The statistical significance for each comparison is analyzed by *t*-test (****, *P*<0.0001). (**B).** The percentage of introns and genes with the three marked intron retention levels in *Fgprp4* compared to the wild type. (**C)**. Introns that were increased in intron retention over 2-fold in *Fgprp4* in both replica experiments. (**D).** Intron splicing defects in the labelled genes were verified by RT-PCR with primers flanking the introns with reduced splicing efficiency (marked with *) in the *Fgprp4* mutant. Lanes 1–3 were PCR results with the genomic DNA, cDNA of PH-1, and cDNA of *Fgprp4*, respectively. The sizes of amplified bands are labelled on the side.

To determine the defects of *Fgprp4* in intron splicing, RNA samples were isolated from aerial hyphae of 9-day-old PDA cultures for RNA-seq analysis. RNA-seq data from two independent experimental replicates were obtained and analyzed. Among the total of 13,321 genes in the *Fusarium graminearum* genome [20], 10,268 have at least one intron and the average intron size is 83 bp. In our RNA-seq data, the expression of 8,028 genes (CPM≥10) was detected in both replicates and 6,359 of them have introns. Although deletion of *FgPRP4* did not completely block intron splicing, the level of retained introns (un-spliced introns) was significantly higher in the mutant than in the wild type (P<0.0001, *t*-test) (Fig 3A). In comparison with the wild type, over 38% of the introns in 47% of the genes with detectable transcripts were increased in intron retention over 2-fold in *Fgprp4* (Fig 3B). Approximately 76% of them (7,837) were identified in both RNA-seq data (Fig 3C), confirming that retention of these introns was related to *FgPRP4* deletion. A third of these introns had over 4-fold reduction in splicing efficiency in both replicates. Nevertheless, splicing of approximately 60% of the predicted introns was not significantly affected (<2-fold) by *FgPRP4* deletion (Fig 3B). Therefore, *FgPRP4* is not essential for intron splicing but it affects splicing efficiency.

Based on GO analysis, genes with over 4-fold reduction in splicing efficiency in the mutant belong to various functional categories, which may contribute to its pleiotropic phenotype. A number of them are known to be functionally related to DNA recombination and repair (S1 Table) based on the functions of their yeast orthologs, including the *FgPHR1* (FGSG_00797), *FgNHP6A* (FGSG_00385), and *FgEAF1* (FGSG_05512) genes that were confirmed to be reduced in splicing efficiency in the *Fgprp4* mutant by RT-PCR analysis (Fig 3D). Therefore, the *Fgprp4* mutant may be compromised in DNA repair.

### Introns affected by *FgPRP4* deletion tend to be longer than unaffected ones

We then compared sequences of the introns that were not affected by *FgPRP4* deletion with those with over 4-fold reduction in splicing efficiency in the mutant. No differences were identified in the sequences of the branch point (BP), 5’ss, and 3’ss (S6 Fig). However, introns with reduced splicing efficiency in the *Fgprp4* mutant tend to be longer (p<0.001) than introns unaffected by *FgPRP4* deletion (S7A Fig), mainly due to longer distance between the BP and 5’ss sequences (S7B Fig). We also noticed that genes with intron splicing efficiency affected by *FgPRP4* deletion tend to have fewer introns that those unaffected in the *Fgprp4* mutant (S7C Fig). Because it is not directly involved in the recognition of 5’ss, BP, and 3’ss sequences, Prp4 likely affects intron splicing by interacting with other spliceosome proteins such as Prp8 [21] or phosphorylation of its substrates in *F*. *graminearum*.

### Spontaneous suppressors of the *Fgprp4* mutant

**Fig 4:**
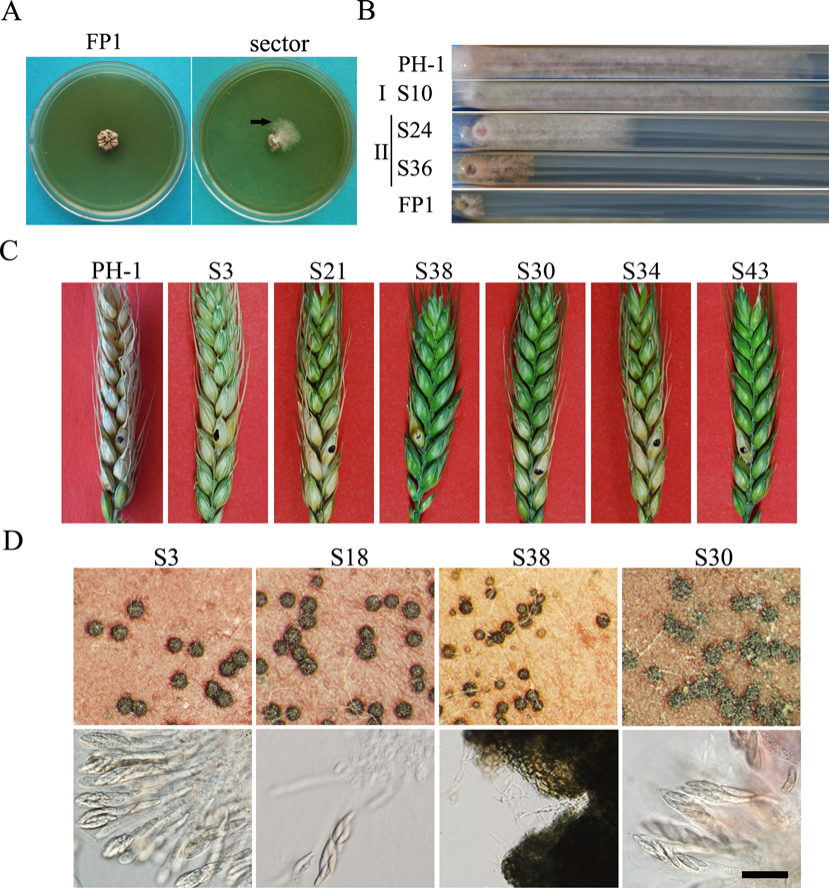
Spontaneous suppressors of the *Fgprp4* mutant. (**A)**. PDA cultures of the *Fgprp4* mutant (FP1) strains after incubation for two weeks. Fast growing sectors were marked with arrows. (**B).** CM cultures of representative type I and type II suppressor mutants grew in race tubes for 14 days. (**C)**. Flowering wheat heads were drop inoculated with conidia of the wild type PH-1 and marked *Fgprp4* suppressor strains. Typical wheat heads were examined 14 dpi. (**D).** Perithecia and asci produced by suppressor strains S3, S18, S38, and S30. Lower panels show asci and ascospores released from cracked perithecia. Bar = 50 μm.

The *Fgprp4* mutant was not stable. Approximately 10% of *Fgprp4* cultures produced fast-growing sectors after incubation for 2 weeks (Fig 4A). We randomly collected 49 subcultures of spontaneous sectors and categorized them into two types based on their growth rate and colony morphology (Fig 4B). Thirty two type I suppressor strains (>65%) had similar growth rate and colony morphology with the wild type. The other 17 type II suppressors grew slower than the wild type but faster than *Fgprp4* (Fig 4B).

For the 32 type I suppressors, we also assayed their defects in conidiation, sexual reproduction, and plant infection (S2 Table). Twenty four of them were still defective in plant infection (Fig 4C). The other 8 were pathogenic on wheat heads but still impaired in sexual reproduction (Fig 4D) or conidiation. (S2 Table). These results indicate that none of these suppressor strains were fully rescued in the defects of *Fgprp4*.

We selected two type I suppressor strains, S2 and S47, for RNA-seq analysis. In comparison with the original *Fgprp4* mutant, only 74.3% and 34.7% of the introns with over 8-fold splicing deficiency were recovered in splicing efficiency in S2 and S47 (S8 Fig), respectively. Therefore, these spontaneous suppressor strains may be not fully recovered in splicing efficiency for all the introns that were affected in the *Fgprp4* mutant. *FgPRP4* must be important for proper regulation of intron splicing and expression of various genes involved in different biological processes.

### Identification of suppressor mutations in components of the U4-U6.U5 complex

To identify suppressor mutations, we sequenced 10 genes orthologous to the known components of the U4/U6 and U4/U6.U5 tri-snRNPs [2,3] amplified from 18 type I and 2 type II suppressor strains (Table 2). Whereas 11 of them had no mutations in these candidate genes, 9 type I suppressor strains had mutations in the *FgPRP6* (FGSG_10242), *FgPRP31* (FGSG_01299), *FgPRP8* (FGSG_02536), and *FgBRR2* (FGSG_01210) genes (Table 2). However, we failed to identify mutations in the rest 11 suppressor strains, suggesting that suppressor mutations may occur in other FgPrp4-targets or tri-snRNP components.

**Table 2:** Candidate Prp4-target genes sequenced in the selected suppressor strains.

	Fg10242	Fg01210	Fg01299	Fg02536	Fg04292	Fg00884	Fg13556	Fg06849	Fg02648	Fg06996
Sc	*PRP6*	*BRR2*	*PRP31*	*PRP8*	*PRP3*	*Cpr1*	*Snu114*	*prp28*	*-*	*Dib1*
Sp	*PRP1*	*SPP41(brr2)*	*PRP31*	*SPP42*(*CSF6*)	*PRP3*	*cyp3*	*cwf10*	*prp28*	*spf38*	*dim1*
Human	*102K*, *PRPF6*	*200K*, *SNRNP200*	*61K*, *PRPF31*	*220K*, *PRPF8*	*90K*, *PRPF3*	*PPIH*, *CypH*	*hSnu114*, *116K*, *EFTUD2*	*prp28*, *100K*, *DDX23*	*40K*, *SNRNP40*	*hDib1*, *15K*, *TXNL4A*
S2	-	-	R464*	-	-	-	-	-	-	-
S3	-	-	-	-	-	-	-	-	-	-
S5	-	-	-	-	-	-	-	-	-	-
S9	-	-	-	-	-	-	-	-	-	-
S10	-	-	-	-	-	-	-	-	-	-
S17	-	-	L532P	-	-	-	-	-	-	-
S19	-	-	-	-	-	-	-	-	-	-
S21	-	-	-	-	-	-	-	-	-	-
S22	R230C	-	-	-	-	-	-	-	-	-
S25	-	-	-	-	-	-	-	-	-	-
S27	-	-	-	-	-	-	-	-	-	-
S28	-	-	-	-	-	-	-	-	-	-
S30	-	G308E	-	-	-	-	-	-	-	-
S33^**^	-	-	-	-	-	-	-	-	-	-
S34	-	-	-	D1153G	-	-	-	-	-	-
S36^**^	-	-	-	-	-	-	-	-	-	-
S39	△E308	-	-	-	-	-	-	-	-	-
S43	-	-	-	E1429K	-	-	-	-	-	-
S46	△E308	-	-	-	-	-	-	-	-	-
S47	R230H	-	-	-	-	-	-	-	-	-

-, sequenced but no changes found

*, stop codon

^**^, type II suppressor

### G308E mutation in *FgBRR2* is the same to A311E mutation in Spp41 of *S*. *pombe*

**Fig 5:**
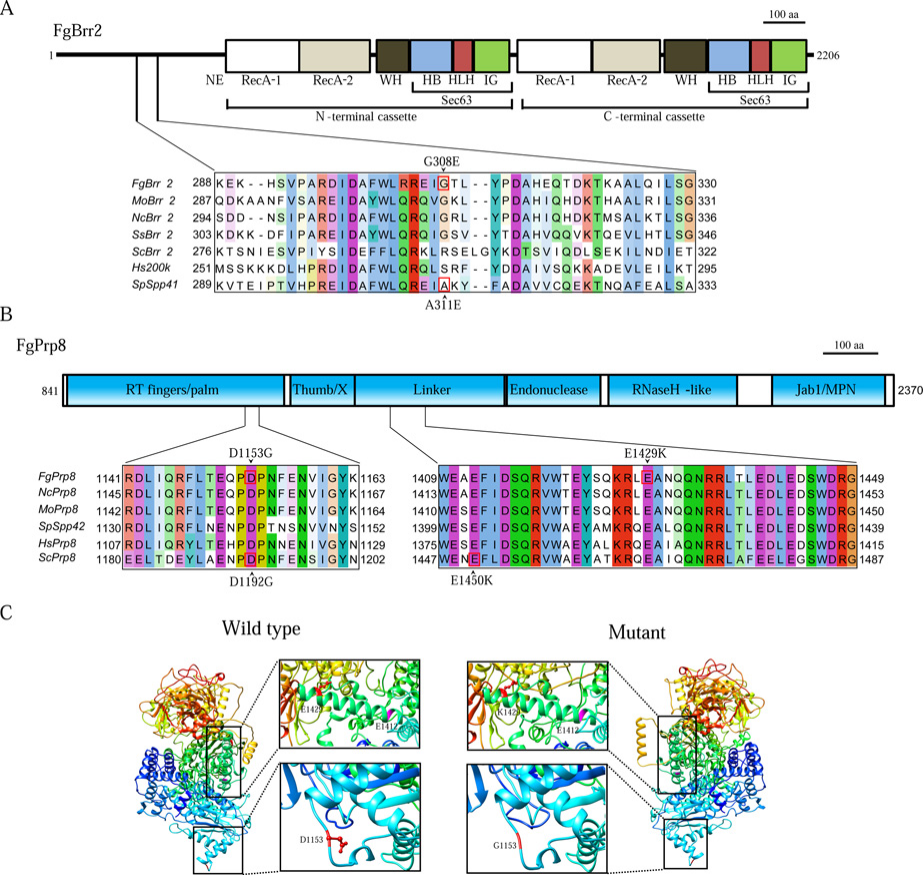
Suppressor mutations in *FgBRR2* and *FgPRP8*. (**A)**. Schematic drawing of the FgBrr2 protein and alignment of the marked region with its orthologs from *M*. *oryzae* (Mo), *N*. *crassa* (Nc), *Sclerotina sclerotiorum* (Ss), *S*. *cerevisiae* (Sc), *S*. *pombe* (Sp), and human (Hs). G308 of FgBrr2 and A311 of Brr2 (Sp) were boxed with red line. Conserved domains of the N- and C-terminal helicase cassettes, G308E mutation in FgBrr2, and A311E in SpSpp41 were labelled. (**B)**. The predicted domain structure of FgPrp8 and sequence alignment of its marked regions with its orthologs from other fungi and humans. (**C)**. 3-D modeling of FgPrp8^846-2370^. The regions with suppression mutations (marked with boxes) were magnified on the right to show the differences between D and G at 1153 or E and K at 1429 in the side chains. E1412 (purple) is in the same cleft with E1429.

In suppressor S30, the G308E mutation was identified in the *FgBRR2* gene (FGSG_01210). G308 is located in the long N-terminal region of Brr2 that has no known motifs but is required for the *in vitro* helicase activity [22]. Sequence alignment showed that G308 of FgBrr2 is at the same position with A311 of Spp41 (Fig 5A). In *S*. *pombe*, the A311E mutation in *spp41* is known to suppress the temperature sensitive *prp4-73* mutant [3]. Therefore, the G308E and A311E mutations that changed a neutral amino acid residue (G or A) to a charged one (E) must have similar effects on the structure and function of the Brr2 helicase.

### D1153G mutation in *FgPRP8* is equivalent to D1192G mutation in *PRP8* of *S*. *cerevisiae*

Two suppressor mutations, D1153G and E1429K (Table 2) were identified in FGSG_02536 that is orthologous to *S*. *cerevisiae PRP8* and *S*. *pombe spp42*. Sequence alignment revealed that both D1153 and E1429 are well conserved in Prp8 orthologs (Fig 5B). D1153 of FgPrp8 is at the same position with D1192 of yeast Prp8, which is in the RT fingers/palm domain. In *S*. *cerevisiae*, the D1192G mutation is a suppressor of the U4-cs1 (cold sensitive) mutant that is defective in U4/U6 unwinding due to a mutation in the U4 RNA [23]. In *F*. *graminearum*, the same D to G mutation in *FgPRP8* suppressed the growth defects of *Fgprp4*, further indicating the role of FgPrp4 in the activation of B-complex and U4-U6 unwinding.

The E1429K mutation occurs in the linker region (Fig 5B). Structural modeling based on yeast Prp8 showed that E1429 and E1412 (= E1450 of yeast Prp8) of FgPrp8 are in the same α-helix that is involved in the formation of the catalytic cavity binding to pre-mRNA (boxed in Fig 5C). E1429K mutation in FgPrp8 may have similar effects with E1450K mutation in yeast on the interaction of Prp8 with the RNA catalytic core.

### Three suppressor mutations occurs near conserved Prp4-phosphorylation sites of FgPrp6

**Fig 6:**
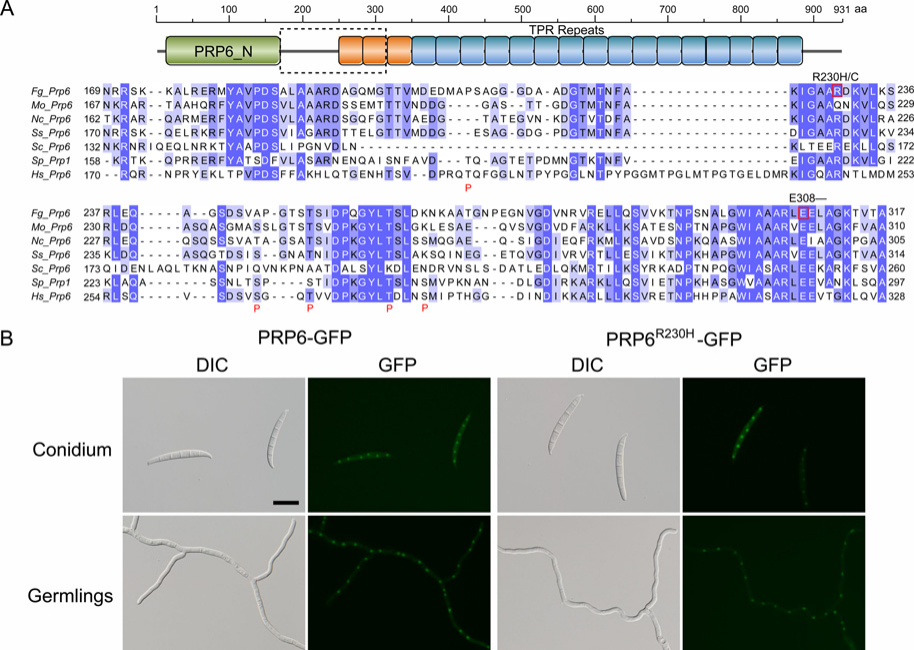
Suppressor mutations in *FgPRP6*. (**A)**. Schematic drawing of the FgPrp6 protein structure showing the PRP6_N domain and 19 TRR repeats. The lower panel shows sequence alignment of the marked region of FgPrp6 with its orthologs from other fungi and humans. Suppressor mutations identified in FgPrp6 were labelled on the top. The putative hPrp4-phosphorylation sites in hPrp6 were boxed and marked with the letter P underneath. (**B)**. Assays for the localization of FgPrp6- and FgPrp6^R230H^-GFP fusion proteins. In transformants expressing the *FgPRP6*-GFP or *FgPRP6*^R230H^-GFP construct, fluorescence signals were primarily observed in the nucleus. The R230H mutation had no obvious effects on the expression and localization of FgPrp31. Bar = 20 μm.

Four suppressor strains had mutations in the ortholog of *S*. *cerevisiae PRP6* (= Prp1 of *S*. *pombe*). The FgPrp6 protein has an N-terminal PRP6_N domain and 19 tetratricopeptide repeats (TPRs). Whereas strains S39 and S46 had the same △E308 mutation, suppressor strains S47 and S22 had R230 changed to H and C, respectively (Fig 6A). In humans, five hPrp4-phosphorylation sites have been identified in the linker region of hPrp6 between the PRP6_N domain and TPR repeats [11]. Sequence alignment showed that two of them, T252 and T261, are conserved in FgPrp6 and its orthologs from other filamentous fungi (Fig 6A). Whereas R230 is in the linker region, E308 is in the first TPR repeat and not far away from the conserved Prp4-phosphorylation sites (Fig 6A). The R230C/H and △E308 mutations may have similar effects on FgPrp6 functions as phosphorylation by FgPrp4 in *F*. *graminearum*.

Arginine methylation is known to affect the nucleocytoplasmic localization of the hnRNP protein A2 [24] and the RNA helicase A [25]. The suppressor mutation in site R230H/C of FgPrp6 is located in a putative non-GAR methylarginine motif GXXR [26,27] that is conserved between FgPrp6 orthologs from filamentous fungi, humans, and *S*. *pombe* (Fig 6A). This non-GAR methylarginine motif is not conserved in Prp6 of *S*. *cerevisiae* (Fig 6A), which lacks Prp4 kinase. To determine whether mutations at R230 will interfere with its subcellular localization, we generated the *FgPRP6*- and *FgPRP6*^R230H^-GFP fusion constructs and transformed them into the wild-type strain. In the resulting transformants, GFP signals were mainly observed in the nucleus (Fig 6B). No obvious difference was observed in the strength or localization of GFP signals between the *FgPRP6*- and *FgPRP6*^R230H^-GFP transformants (Fig 6B). Therefore, R230H mutation had no effect on the localization of FgPrp6.

### Two mutations in the C-terminal region of FgPrp31 rescue the *Fgprp4* mutant

**Fig 7:**
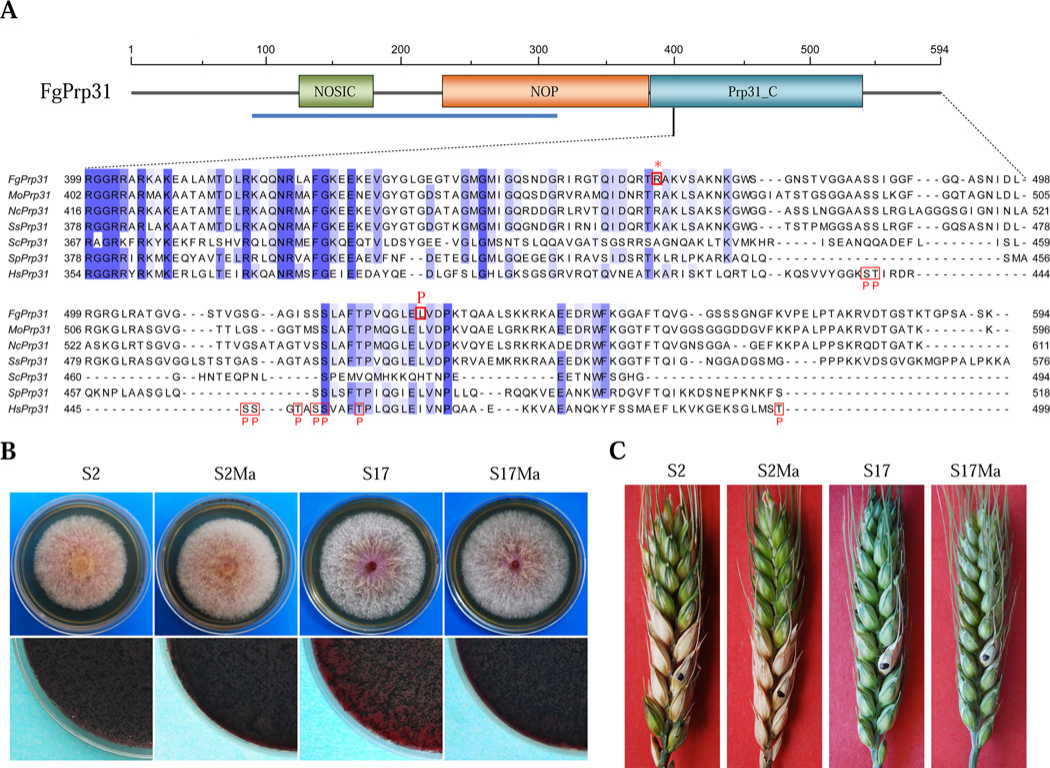
Suppressor mutations in *FgPRP31*. (**A)**. Schematic drawing of FgPrp31 (with the NOSIC, NOP, and PRP31_C domains) and sequence alignment of its C-terminal region with its orthologs from *M*. *oryzae* (Mo), *N*. *crassa* (Nc), *S*. *sclerotiorum* (Ss), *S*. *cerevisiae* (Sc), *S*. *pombe* (Sp), and human (Hs). Blue line indicates the region with known 3-D structures in hPrp31. The R464* and L532P mutations were labelled on the top. Putative hPrp4-phosphorylation sites in hPrp31 are boxed and labelled with the letter P. (**B).** Three-day-old PDA cultures (upper row) and 2-week-old mating plates (lower row) of suppressor strains S2 and S17, *Fgprp4 FgPRP31*^R464*^ transformant S2Ma, and *Fgprp4 FgPRP31*^L532P^ transformant S17Ma. (**C)**. Flowering wheat heads inoculated with the same set of strains examined 14 dpi.

In suppressor mutant S17, the L532P mutation was identified in *FgPRP31* (FGSG_01299). Interestingly, the non-sense mutation at R464 in suppressor S2 resulted in the truncation of the C-terminal 130 aa residues of FgPrp31, including L532 (Fig 7A). In RNA-seq data with suppressor strain S2, the *FgPRP31* transcripts also had the G1392A mutation that caused the change of R464 (CGA) to a stop codon (UGA). Whereas the NOSIC and NOP domains (spanning the 93–368 aa region) are well-conserved and known to interact with Prp6 and the U4 RNA, the R464* and L532P suppressor mutations occurred in or after the less-conserved PRP31_C (Fig 7A).

Although the exact phosphorylation site or function is not clear, nine hPrp4-phosphorylation sites have been identified in hPrp31 by phosphoproteomics analysis [11]. Five of them, S485, S486, S520, S521, and T525 are conserved in FgPrp31 (Fig 7A). The nonsense mutation at R464 eliminated all of these putative Prp4-phosphorylation sites in FgPrp31. These data suggest that the C-terminal region of FgPrp31 likely plays a negative role in B-complex activation, possibly by inhibitory binding to its own N-terminal region or other Prp31-interacting proteins. Phosphorylation by FgPrp4 in the phosphorylation or modulation region may result in conformational changes and release the inhibitory self-binding.

### Validation of the R464* and L532P mutations in *FgPRP31*

We selected *FgPRP31* for further characterization because of interesting features of the R464* truncation mutation. The geneticin resistant *FgPRP31*^R464*^ and *FgPRP31*^L532P^ gene replacement constructs were generated and co-transformed with the hygromycin-resistant *FgPRP4* knockout cassette [18] into protoplasts of PH-1. Transformants resistant to both hygromycin and geneticin were screened by PCR for deletion of *FgPRP4*. In the resulting *Fgprp4* mutants, the replacement of endogenous *FgPRP31* with the *FgPRP31*^R464*^ or *FgPRP31*^L532P^ mutant allele was confirmed by PCR amplification and sequencing analysis. Similar to suppressor strains S2 and S17, the *Fgprp4*/*FgPRP31*^R464*^ and *Fgprp4*/*FgPRP31*^L532P^ transformants were normal in growth rate and sexual reproduction (Fig 7B) but still defective in plant infection (Fig 7C). Therefore, the R464^*^ and L532P mutations are directly responsible for the recovery of growth rate in suppressor strains S2 and S17.

### Identification of suppressor mutations in suppressor S3 by whole genome sequencing

Because mutations were not identified in 11 type I suppressors that were analyzed, we selected suppressor S3 for whole genome sequencing analysis. After aligning the sequences of S3 (approximately 50 coverage) generated by Illumina Hi-seq with the genome sequence of PH-1, the C to A mutation at 305 was identified in FGSG_11793, which is orthologous to yeast *MSL1*, a U2B component of the U2 SNP [28]. The resulting Q to K mutation occurred at the Q86 residue that is conserved in *MSL1* orthologs from filamentous fungi (S9 Fig). The Q86K mutation is in the predicted RNA recognition motif (RRM) domain (S9 Fig) and will likely affect its interaction with pre-mRNA or other components of sn-RNP during B-complex activation.

### Phosphorylation of S289 in FgPrp4 is important for its function

**Fig 8:**
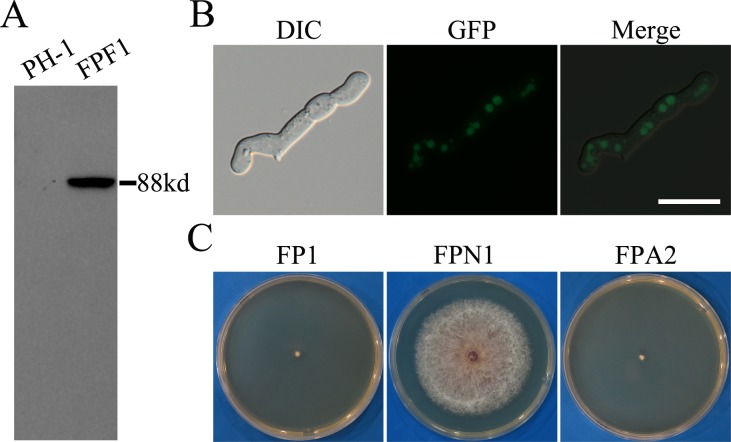
The S289 mutation affected the function but not localization of FgPrp4. (**A).** Western blots of total proteins isolated from the wild type (PH-1) and the *FgPRP4*-3xFLAG transformant were detected with an anti-3xFLAG antibody. (**B).** 12 h germlings of the *Fgprp4/FgPRP4*^S289A^ transformant FPA2 were examined by DIC and epifluorescence microscopy. Bar = 20 μm. **(C).** Three-day old PDA cultures of the *Fgprp4* mutant (FP1), complemented transformant (FPN1), and *Fgprp4/FgPRP4*^S289A^ transformant (FPA2).

To determine whether FgPrp4 kinase itself is activated by phosphorylation, we generated the *FgPRP4*-3xFLAG construct and transformed it into PH-1. The resulting transformant FPF1 (Table 1) had the expected 88-KD Prp4-3xFLAG fusion protein band on western blots detected with the anti-FLAG antibody (Fig 8A). To assay FgPrp4 phosphorylation, total proteins isolated from the *FgPRP4*-3xFLAG transformants were incubated with anti-FLAG beads. Proteins eluted from anti-FLAG beads were treated with trypsin and enriched for phosphopeptides with the PolyMac approach as described [29]. The resulting peptides were analyzed by MALDI-TOF/TOF MS analysis. In three independent biological replicates, phosphorylation of S289 was detected in the peptide AAS^289^PASTLP of FgPrp4.

Because S289 was the only phosphorylation site identified in *FgPRP4*, we generated the *FgPRP4*^S289A^-GFP mutant allele and transformed it into the *Fgprp4* mutant. The resulting *Fgprp4/FgPRP4*^S289A^ transformant FPA2 (Table 1) had GFP signals in the nucleus (Fig 8B) but, like the original mutant, displayed severe growth and conidiation defects (Fig 8C), indicating that *FgPRP4*^S289A^ failed to complement the *Fgprp4* mutant in growth and conidiation. Therefore, phosphorylation at S289 is essential for FgPrp4 functions. It is possible that FgPrp4 is activated by phosphorylation at S289 by itself or other protein kinases for spliceosome activation in *F*. *graminearum*.

## Discussion

Among all the spliceosome components, Prp4 is the only protein kinase and it is conserved in humans, plants, and *S*. *pombe* [21,30]. Interestingly, all the sequenced Saccharomycotina species except *Y*. *lipolytica* lack a distinct Prp4 ortholog (S1 Fig). Whereas *S*. *cerevisiae* has only 376 introns, *Y*. *lipolytica*, a dimorphic yeast, has over 1,500 introns [31]. Because lower fungi such as *Rhizopus oryzae* and *Batrachochytrium dendrobatidis* have this kinase gene, Saccharomycotina species may have lost the *PRP4* ortholog after massive intron loss during evolution [17,32].

In *F*. *graminearum*, the *Fgprp4* mutant was viable although it had severe growth defects. To our knowledge, null mutants of the Prp4 kinase have not been reported in any other eukaryotic organisms except in *N*. *crassa*, in which the putative *stk*-57 mutant deleted of the *PRP4* ortholog (NCU10853) generated in a large-scale protein kinase gene knockout study had no defects in hyphal growth, asexual reproduction, and sexual development but could not be purified by isolation of ascospores [33]. Because of its striking difference from the *Fgprp4* mutant, we obtained the putative *stk*-57 mutant (stock number FGSC17970) from Fungal Genetics Stock Center (www.fgsc.net) and conducted PCR analyses. Both the *STK*-57 kinase gene and the hygromycin-resistant marker could be amplified in this putative knockout mutant (S10 Fig). Furthermore, we failed to amplify any PCR products with the anchor primers that were designed to amplify the upstream and downstream fragments resulted from gene replacement events (S10 Fig). These results indicate that this putative *stk*-57 knockout mutant was not a true mutant but likely an ectopic transformant.

Considering the fact that many essential genes have introns in *F*. *graminearum*, the viability of *Fgprp4* mutant suggests that deletion of *FgPRP4* does not block spliceosome activation and intron splicing. This hypothesis was confirmed by RNA-seq data. FgPrp4 kinase is not essential for RNA splicing but it regulates splicing efficiency. Consistent with its pleiotropic defects, splicing efficiency of introns in over 39% of the *F*. *graminearum* genes involved in various physiological and developmental processes were reduced significantly in the *Fgprp4* mutant. Although no unique 5’ss, BP, and 3’ss sequences were identified in introns affected in the mutant, we noticed that splicing of larger introns with longer distance between the BP and 5’ss sequences is more sensitive to *FgPRP4* deletion. In addition, intron splicing efficiency in the *Fgprp4* mutant was not related to predicted gene functions. In fact, it is often that the splicing efficiency was only affected by *FgPRP4* deletion for some but not all the introns in the genes with multiple introns in *F*. *graminearum*. Furthermore, we noticed that the position of introns in mRNA has no effects on intron splicing affected by *FgPRP4* deletion. The budding yeast has approximately 300 genes with small introns although it lacks the Prp4 ortholog. Among 136 of them with orthologs in *F*. *graminearum*, only two of them had normal intron splicing efficiency in the *Fgprp4* mutant. Therefore, the function and evolutional relationship of genes have no effect on whether intron splicing was affected or not by deletion of *FgPRP4* in *F*. *graminearum*.

The *Fgprp4* mutant was unstable and produced fast growing sectors. Our RNA-seq and RT-PCR results showed that deletion of *FgPRP4* resulted in splicing defects in a number of genes important for DNA recombination and repairing, which may be responsible for the production of spontaneous suppressors. Among the 49 sectors we isolated, over 60% were fully recovered in the growth rate and colony morphology, the others grew faster than the original mutant but still slower than the wild type, and may had additional defects in aerial hyphal growth or colony pigmentation, indicating that suppressor mutations may occur in different genes. Even for spontaneous suppressors with the wild-type growth rate and colony morphology, none of them were normal in all the other phenotypes assayed, including virulence, conidiation, and sexual reproduction. Therefore, although suppressor mutations suppressed the defects of *Fgprp4* mutant in vegetative growth, they failed to rescue all the other defects associated with *FgPRP4* deletion. This observation may explain why *F*. *graminearum* still keep the *FgPRP4* gene although suppressor mutations occur at such a high frequency in its deletion mutant.

**Fig 9:**
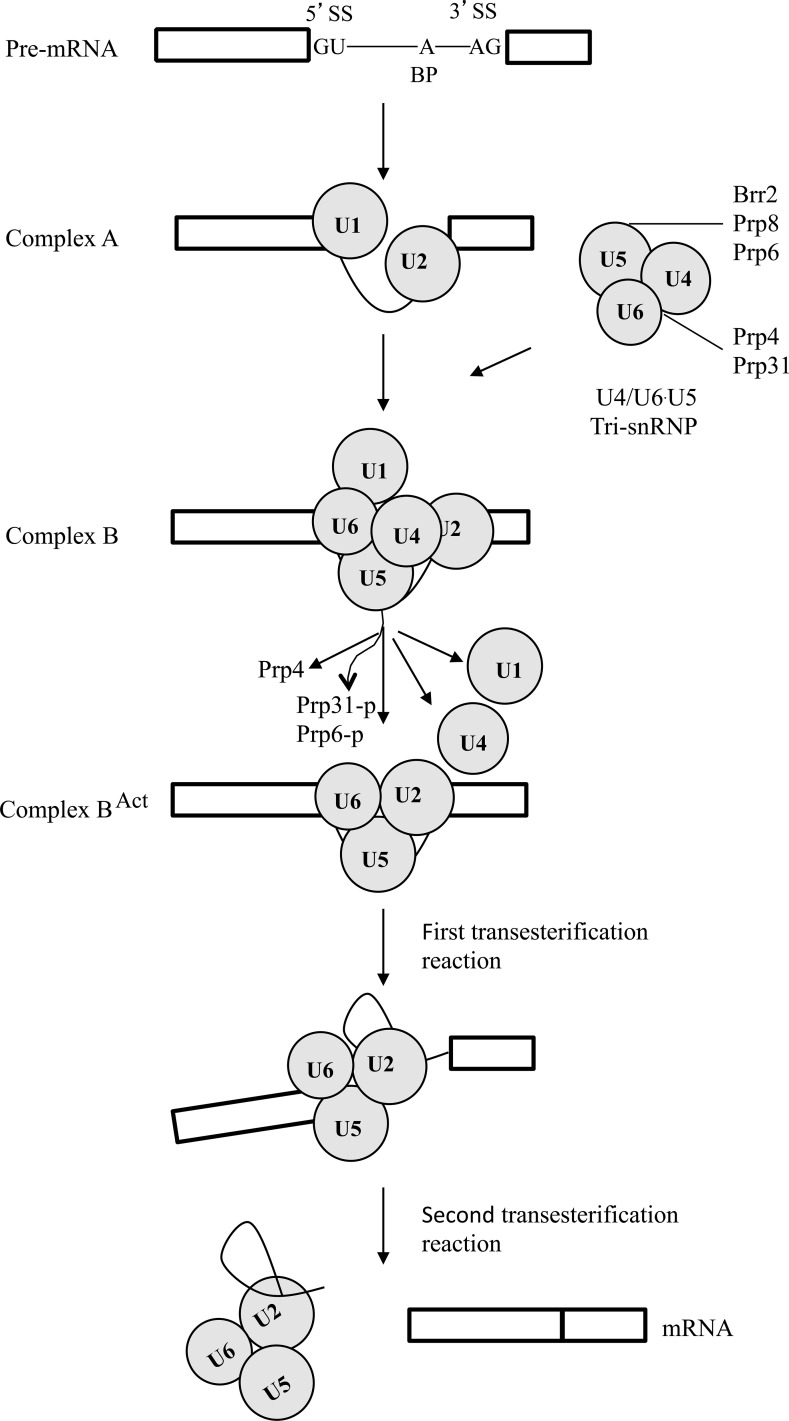
Schematic draw of the pre-mRNA splicing processes and components of tri-snRNP related to this study. Exons and one intron are represented by boxes and solid line, respectively. Base pairing of U1 to 5’ss and recognition of BP by U2 (formation of complex A) are followed by the integration of preformed U4/U6-U5 tri-snRNP to form complex B. Whereas Prp4 and Prp31 are components of U4/U6 snRNP, Brr2, Prp8, and Prp6 are components of U5 snRNP. Phosphorylation of Prp6 and Prp31 by Prp4 is associated with the activation of B-complex (complex B^act^). U1, U4, Prp4, Prp6, and Prp31 are released from the activated spliceosome that catalyzes two sequential transesterifications reactions for intron splicing.

Prp6, Prp8, Prp31, and Brr2 are key components of the U4/U6-U5 tri-snRNP [34,35] (Fig 9). Suppressor mutations identified in these genes may have similar effects with phosphorylation by Prp4 on the interactions among these tri-snRNP components. In *S*. *pombe*, suppressor mutations of the *prp4-73*^ts^ mutant have been identified in the Brr2 (Spp41) and Prp8 (Spp42) orthologs [3,36]. The G308E mutation of FgBrr2 is the same to A311E of Spp41, changing from a neutral, non-polar residue (G or A) to an acidic, polar one (E). G308 is in the N-terminal region of Brr2 required for the *in vitro* helicase activity [22]. The N-terminal region, RecA-1, and RecA-2 of hBrr2 also may be involved in interacting with hPrp6 [9]. Therefore, G308E and A311E mutations may have similar effects on Brr2 helicase activity or its interaction with Prp6 to suppress *prp4* mutant.

For the G2248D suppressor mutation characterized in *spp42* [36], we failed to identify mutations at the same residue in *FgPRP8*. However, D1153G, one of the two suppressor mutations identified in *FgPRP8*, is the same to D1192G of *PRP8* that could suppress the yeast U4-cs1 mutant [23]. When modeled after the crystal structure of yeast Prp8^885-2413^, D1153 is at the tip of the exposed loop following the RTα12. Interestingly, this region of Prp8 also contains other suppressor mutations of U4-cs1 and suppressor mutations of *brr2-1* [37,38], suggesting its involvement in interaction with Brr2 and other proteins or RNA. The D to E mutation may affect the interaction of Prp8 with other tri-snRNP components. Nevertheless, the D1153E mutation in *FgPRP8* suppressed the *Fgprp4* mutant, indicating that FgPrp4 may play a critical role in U4/U6 unwinding by affecting the interactions between different tri-snRNP components.

Like D1153G, E1429K did not change the overall structure of FgPrp8. In yeast Prp8, the E1450K mutation suppresses the 3’ss mutation [38,39], indicating its involvement in RNA binding. In FgPrp8, E1429 and E1412 (= E1450 of yeast Prp8) are in the same α-helix that is involved in the formation of the catalytic cavity [38]. Because they are in the same cleft and have the same E to K change, the E1429K and E1450K mutations may have similar effects on the catalytic cavity of tri-snRNP and B-complex activation.

In *S*. *pombe*, suppressor mutations of *prp4-73*^ts^ mutant have not been reported in Prp6. In humans, phosphorylation of Prp6 that occurs after the tri-snRNP being integrated into the B-complex may release the inhibition of Brr2 by Prp8 and is important for spliceosomal B-complex activation [3,11]. In this study, we identified four suppressor strains with mutations in the *PRP6* ortholog. In RNA-seq data with suppressor strains, the transcripts of *FgPRP6* had the C690U mutation in suppressor strain S47, which is consistent with the *FgPRP6*^R230H^ mutation detected by DNA sequencing analysis. Because both R230 and E308 are in the proximity of T252 and T261, two conserved Prp4-phosphorylation sites, it is likely that mutations at these two residues have similar effects with phosphorylation by FgPrp4 on FgPrp6 functions.

Among 20 suppressor mutants that were sequenced, we only identified suppressor mutations in nine of them. For the other 11 suppressor strains, none of them had mutations in the candidate tri-snRNP components that were selected for sequencing analysis. Suppressor mutations likely occur in other tri-snRNP components, such as orthologs of Snu13, Snu66, Snu114, Cpr1, Sad1, and Dib1 [40,41]. However, in suppressor S3, the Q86K mutation in *FgMSL1* was identified by whole genome sequencing. To our knowledge, suppressor mutations in *MSL1* orthologs have not been reported in other organisms. In *S*. *cerevisiae*, *MSL1* is an nonessential gene that encodes a U2 snRNP-specific protein [42]. In *Drosophila*, the U2B protein is part of a protein network that is important for splicing accuracy and efficiency [43]. In *F*. *graminearum*, the Q86K mutation suppressed the defects of the *FgPrp4* mutant in growth. It is possible that this mutation in *FgMSL1* may affect the U2-U6 coupling and complex B activation.

Unlike Prp4 in *S*. *pombe*, FgPrp4 has a long N-terminal SR-rich region/domain that is conserved in metazoan Prp4 kinases [44]. Expression of the *FgPRP4*^ΔN310^-GFP allele failed to complement the *Fgprp4* mutant and GFP signals became localized to the cytoplasm instead of the nucleus, indicating that this N-terminal region is important for the function and subcellular localization of FgPrp4 in *F*. *graminearum*. This region contains two putative NLS sequences conserved among the FgPrp4 orthologs. To our knowledge, the NLS sequence responsible for the localization of Prp4 kinases to the nucleus has not been characterized. It will be important to further characterize the function of these two NLS sequences in the N-terminal region of FgPrp4. In humans, a number of other splicing factors, such as SRSF1 and ASF/SF2, also have the N-terminal RS-rich region that may be phosphorylated by SR protein kinases such as CLK and SRPK [45]. In this study, we showed that S289 is a phosphorylation site important for FgPrp4 functions. Because auto-phosphorylation of Prp4 has been reported in humans [44], it will be important to determine whether phosphorylation of S289 is catalyzed by FgPrp4 itself or other protein kinases.

## Materials and Methods

### Culture conditions and plant infection assays

The wild-type strain PH-1, *Fgprp4* mutants, and all the transformants generated in this study were routinely cultured on potato dextrose agar (PDA) [46] or complete medium (CM) at 25^°^C and preserved in 20% glycerol at -80°C [47]. Growth rate, conidiation, and sexual reproduction were assayed as described [18]. Protoplasts prepared from 12 h germlings were used for PEG-mediated transformation [48]. For infection assays, flowering wheat heads of cultivar XiaoYan 22 were drop-inoculated with 10 μl of conidium suspensions (2.0×10^5^ conidia/ml) as described [49]. Scab symptoms were examined 14 days post-inoculation (dpi).

### qRT-PCR analysis

RNA was isolated with the TRIzol reagent (Invitrogen) from conidia, 12 h germlings, perithecia at 10 days post-fertilization, and infected wheat heads collected at 7 dpi as described [50,51]. For qRT-PCR analysis, first-strand cDNA was synthesized with the Fermentas 1st cDNA synthesis kit (Hanover) following the instructions provided by the manufacturer. The β-tubulin gene FGSG_06611 of *F*. *graminearum* was used as the internal control [52]. The mean and standard deviation were calculated with data from three biological replicates.

### Generation of the *Fgprp4/FgPRP4*, *Fgprp4/FgPRP4*^ΔN310^*-*GFP, *Fgprp4/FgPRP4*^S289A^, and *Fgprp4/FgPRP4-*GFP transformants

For complementation assays, the *FgPPR4* gene was cloned into pFL2 [48] by gap repair [53]. The resulting *FgPRP4* construct carrying the geneticin-resistant marker was transformed into the *Fgprp4* mutant FP1. The same gap repair approach was used to generate the *FgPRP4-*GFP, *Fgprp4/FgPRP4*^S289A^, and *FgPRP4*^ΔN310^*-*GFP construct with primers showed in S3 Table. The resulting constructs were confirmed by sequencing analysis and transformed into protoplasts of FP1 to generate the complemented transformants.

### Spontaneous suppressors of the *Fgprp4* mutant

Fast-growing sectors of the *Fgprp4* mutant were transferred with sterile toothpicks to fresh PDA plates. After single spore isolation, each sub-cultures of spontaneous suppressors were assayed for defects in growth, differentiation, and plant infection [18]. To identify suppressor mutations in the candidate tri-snRNP components, PCR products amplified with primers listed in S3 Table were sequenced at BGI-Beijing. Mutation sites were identified by sequence alignment and confirmed by re-sequencing analysis.

### RNA-seq analysis

Vegetative hyphae of PH-1, *Fgprp4* mutant FP1, S2, and S47 were harvested from 9-day-old PDA colonies formed over sterile dialysis membrane and used for RNA isolation with the TRIzol Reagent (Life technologies, US). Poly(A) mRNA was isolated with the Oligotex mRNA mini kit (Qiagen, Germany). Library construction and sequencing with an Illumina Hiseq 2000 sequencer were performed at Shanghai Biotechnology Corporation (Shanghai, China). For each sample, at least 25 Mb high-quality reads were obtained. The resulting RNA-seq reads were mapped onto the reference genome of *F*. *graminearum* strain PH-1 with the Tophat2 program (ccb.jhu.edu/software/tophat/index.shtml). To filter out weakly expressed genes, only genes with a minimum expression level of 1 count per million were included in the analysis. The intron retention level was defined as the number of reads that aligned to the predicted intron divided by the number of reads aligned to the corresponding transcript.

### RT-PCR analyses

RNA was isolated with the TRIzol Reagent (Life technologies) from vegetative hyphae of PH-1 and the *Fgprp4* mutant. The Fermentas 1^st^ cDNA synthesis kit (Hanover, MD, USA) was used to synthesize the first-strand cDNA following the instruction provided by the manufacturer. The primers used for PCR amplification of the *FgPHR1* (FGSG_00797), *FgNHP6A* (FGSG_00385), and *FgEAF1* (FGSG_05512) genes were listed in S3 Table.

### Identification of phosphorylation sites in FgPrp4

The *FgPRP4*-3xFLAG fusion construct was generated by the gap repair approach by co-transformation of the full-length *FgPRP4* fragment and *Xho*I-digested pFL7 into yeast strain XK1-25 [48]. The resulting fusion construct rescued from Trp^+^yeast transformants was confirmed by sequence analysis and transformed into the wide-type strain PH-1. Geneticin-resistant transformants expressing the fusion constructs were identified by PCR and confirmed by western blot analysis with the anti-FLAG antibody (Sigma). Total proteins isolated from the resulting transformant were incubated with the anti-FLAG M2 beads (Sigma) as described [54]. Proteins eluted from anti-FLAG beads were digested with proteomics grade trypsin (Sigma) and enriched for phosphopeptides with the polymer-based metal ion affinity capture (PolyMAC) as described [29]. Phosphopeptides enriched by PolyMac were analyzed with an ABI 4800 MALDI-TOF/TOF mass spectrometer. Proteome Discoverer (version 1.0; Thermo Fisher Scientific) was used to identify peptide sequences and phosphorylation sites as described [29].

### Sequence comparison and phylogenetic analysis

Multiple alignments of protein sequences were constructed with COBALT (www.ncbi.nlm.nih.gov/tools/cobalt) and manually modified. The analysis of type I and type II functional divergence was performed with the Diverge 3.0 software [55]. Maximum likelihood (ML) phylogenies were estimated with PhyML3.0 assuming 8 categories of γ-distributed substitution rate and SPRs algorithms. For phylogeny of protein sequences, the bestfit model for each datasets selected by ProtTest2.4 [56] was used. The reliability of internal branches was evaluated based on SH-aLRT supports. The 3D-structural model of FgPrp8 was modeled after that of Prp8 in *S*. *cerevisiae* (PDB ID: 3SBT and 2OG4) and displayed with Chimera 1.8.1 [57].

### Whole genome sequencing analysis with suppressor strain S3

To identify mutations in suppressor S3, DNA isolated from 12 h germlings were sequenced by Illumina platform at Shanghai Biotechnology Corporation (Shanghai, China) to 50x coverage with pair-end libraries. The sequence reads were mapped onto reference genome of strain PH-1 by using Bowtie 2.23 [58]. Mutation sites were called by SAMtools with the default parameters. Annotation of the mutation sites was performed with Variant Effect Predictor (VEP) [59].

### Data deposition

RNA-seq data generated in this study were deposited in the NCBI Sequence Read Archive database under the accession code of SRP062439.

## Supporting Information

S1 Fig Phylogenetic tree of FgPrp4 and its orthologs from other fungi. (TIF)

S2 Fig Generation and verification of the *Fgprp4* mutant. (**A**) Schematic draw of the *FgPRP4* gene and gene replacement construct. 1F, 2R, 3F, 4R, 5F, 6R, H850, and H852 are the primers used to generate or verify *FgPRP4* gene replacement mutants. X, *Xho*I. (**B**) Southern blots of genomic DNA of PH-1 (WT) and the *Fgprp4* mutant (*prp4*) digested with *Xho*I were hybridized with a *FgPRP4* fragment amplified with primers 5F/6R (Probe A) or a fragment of the hygromycin-phosphotransferase gene (*hph*) amplified with primers H850/H852.
(TIF)

S3 Fig Three-day old PDA cultures of the wild type (PH-1), and the *Fgprp4*/*FgPRP4*-GFP transformant (FPN1). (TIF)

S4 Fig Alignment of the amino acid sequences of FgPrp4 and its orthologs from humans (Hs), *Magnaporthe oryzae* (Mo), *Neurospora crassa* (Nc), *Aspergillus nidulans* (An), and *S*. *pombe* (Sp). Two predicted nuclear localization sequences (NLS) and the kinase domain are boxed and labelled. P marks putative phosphorylation site in FgPrp4.
(TIF)

S5 Fig Two isoforms of *FgPRP4* transcripts. (**A**). IGV Sashimi plots showing the read numbers and splice junctions of *FgPRP4* transcripts in marked RNA-seq data. **(B).** Schematic draws of *FgPRP4* and its two transcript isoforms. The orange boxes are the kinase domain region. (**C**). qRT-PCR analysis with isoforms A and B of *FgPRP4* transcripts. The relative expression level of isoform A in conidia was arbitrarily set to 1.
(TIF)

S6 Fig **Sequence features of the 5’ss (A), 3’ss (B), and BP (C) of the introns that were significantly affected or not affected by *FgPRP4* deletion in splicing efficiency.** The 5’ss, 3’ss and BP sequences are marked by green rectangles.
(TIF)

S7 Fig Intron features affected by *FgPRP4* deletion. (**A)**. Introns with reduced splicing efficiency in the *Fgprp4* mutant tend to be longer than introns unaffected by *FgPRP4* deletion (P<0.001). (**B)**. The distance between 5’ss and BP but not the distance between BP and 3’ss is longer in introns affected by *FgPRP4* deletion than those not affected. **(C).** Genes with reduced in intron splicing efficiency in the *Fgprp4* mutant tend to have fewer introns than genes not affected by *FgPRP4* deletion. ****, P<0.0001.
(TIF)

S8 Fig Percentage of introns and genes with splicing efficiency recovered in in suppressor strains S2 and S47. (TIF)

S9 Fig Suppressor mutations in *FgMLS1*. Schematic drawing of the FgMls1 protein structure showed the RRM domain and sequence alignment of its RRM domain of other Mls1 orthologs. The Q86K mutation was labelled on the top and the RBD motif was boxed.
(TIF)

S10 Fig PCR analysis with the wild-type and putative *stk*-57 mutant strains of *Neurospora crassa*. (**A**) Schematic draw of the *STK57* gene, hygromycin-phosphotransferase (*hph*) cassette, and the positions/directions of PCR primers. (**B**) PCR analysis with labelled primer pairs with genomic DNA of the wild type (**a**) and putative *stk*-57 mutant (**b**). The expected PCR products amplified by primer pairs SF1/SR2, HF1/HR2, SF3/HR3, and HF4/SR4 were labelled on the side. M, 1 kb DNA Ladder (NEB).
(TIF)

S1 Table Splicing defects in genes related to DNA repair. (DOCX)

S2 Table Defects of the suppressor strains in conidiation, pathogenesis, and sexual reproduction. (DOC)

S3 Table Primers used in the study. (DOC)
