# Supplementary figures and images for: FgPrp4 Kinase Is Important for Spliceosome B-Complex Activation and Splicing Efficiency in Fusarium graminearum
Source: PLoS Genet. 2016 Apr 8;12(4):e1005973. doi: 10.1371/journal.pgen.1005973 (PMC4825928; doi:10.1371/journal.pgen.1005973)

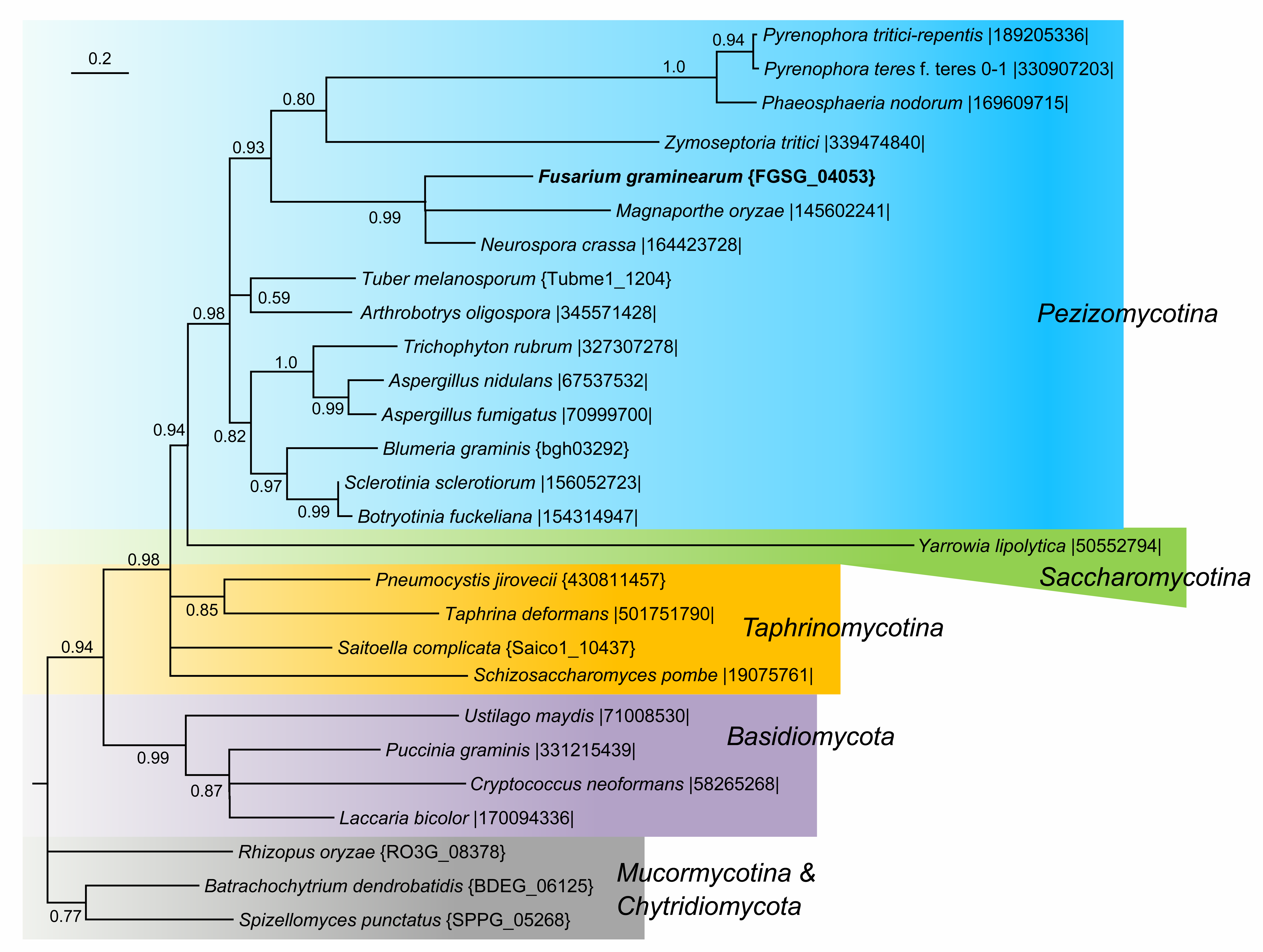

Supplement: S1 Fig — (TIF) [file pgen.1005973.s001.tif]

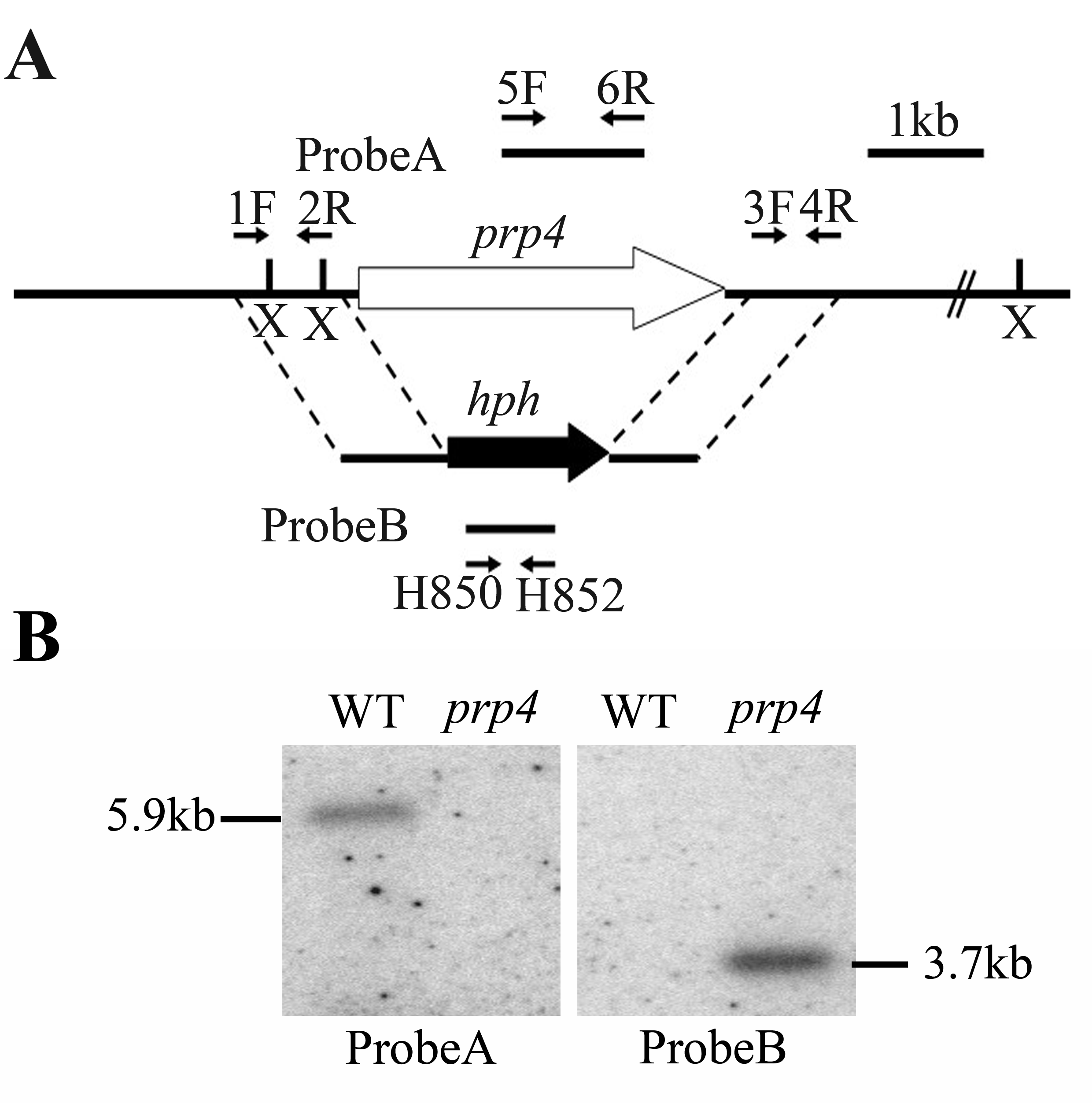

Supplement: S2 Fig — (A) Schematic draw of the FgPRP4 gene and gene replacement construct. 1F, 2R, 3F, 4R, 5F, 6R, H850, and H852 are the primers used to generate or verify FgPRP4 gene replacement mutants. X, XhoI. (B) Southern blots of genomic DNA of PH-1 (WT) and the Fgprp4 mutant (prp4) digested with XhoI were hybridized with a FgPRP4 fragment amplified with primers 5F/6R (Probe A) or a fragment of the hygromycin-phosphotransferase gene (hph) amplified with primers H850/H852. (TIF) [file pgen.1005973.s002.tif]

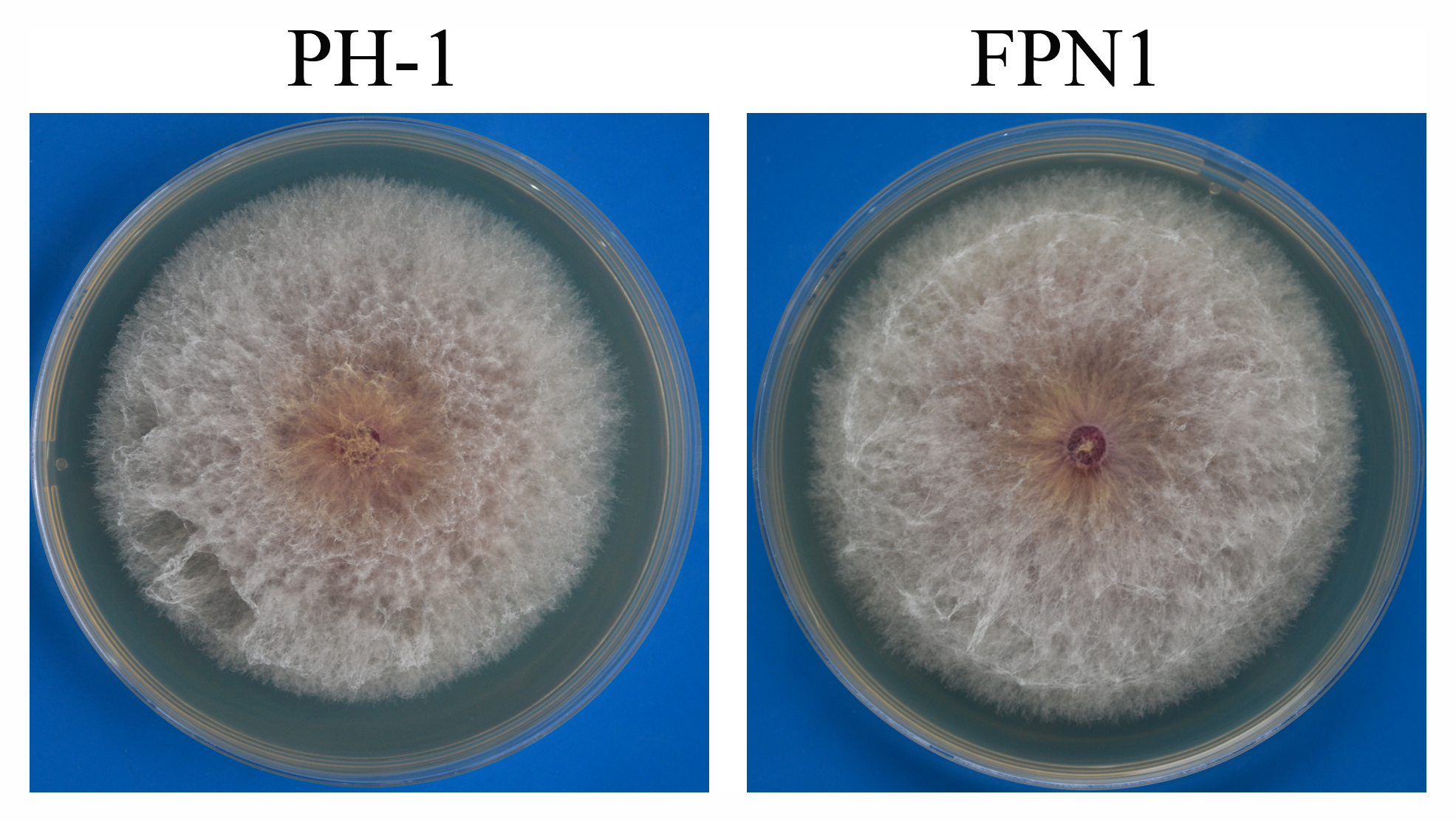

Supplement: S3 Fig — (TIF) [file pgen.1005973.s003.tif]

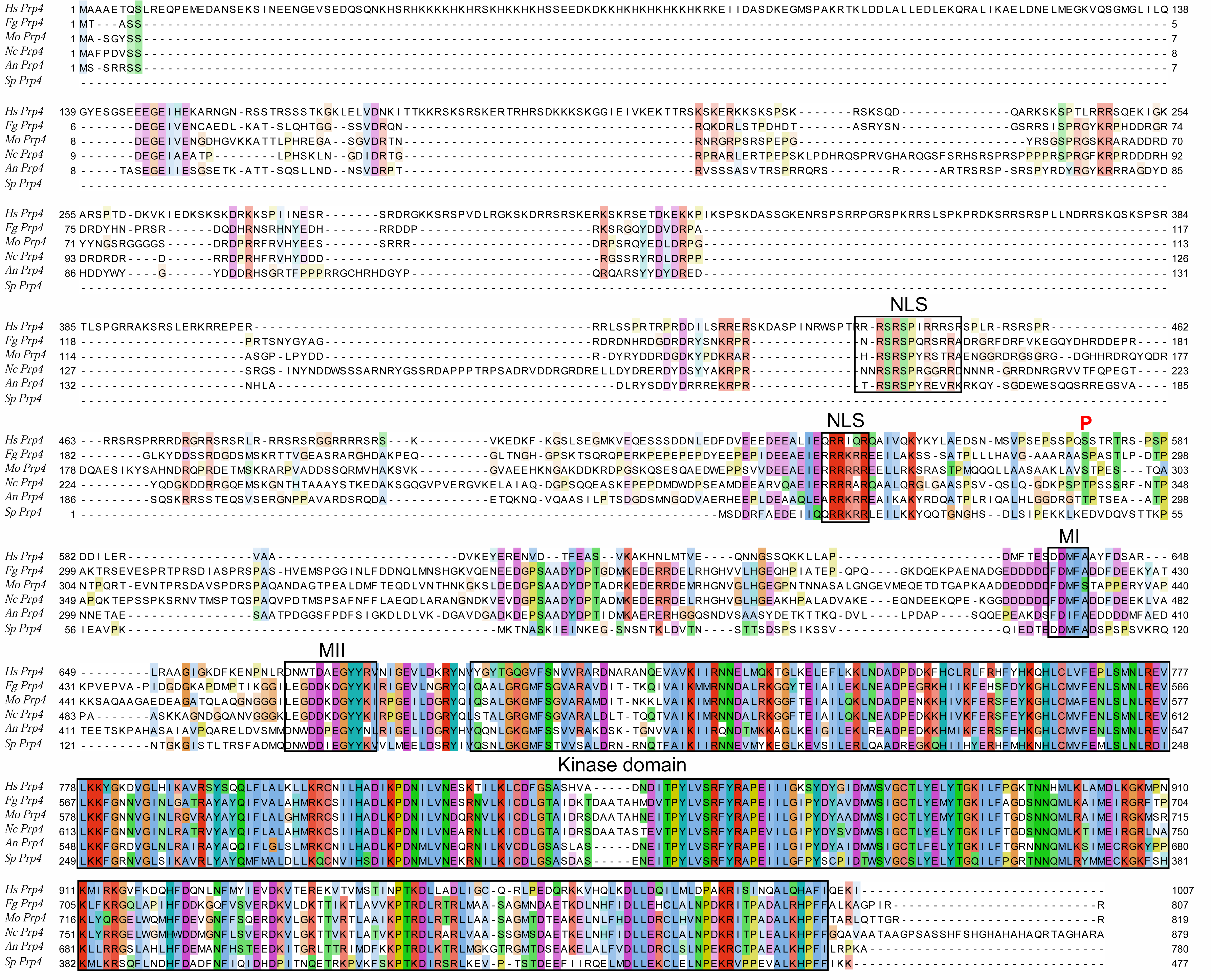

Supplement: S4 Fig — Two predicted nuclear localization sequences (NLS) and the kinase domain are boxed and labelled. P marks putative phosphorylation site in FgPrp4. (TIF) [file pgen.1005973.s004.tif]

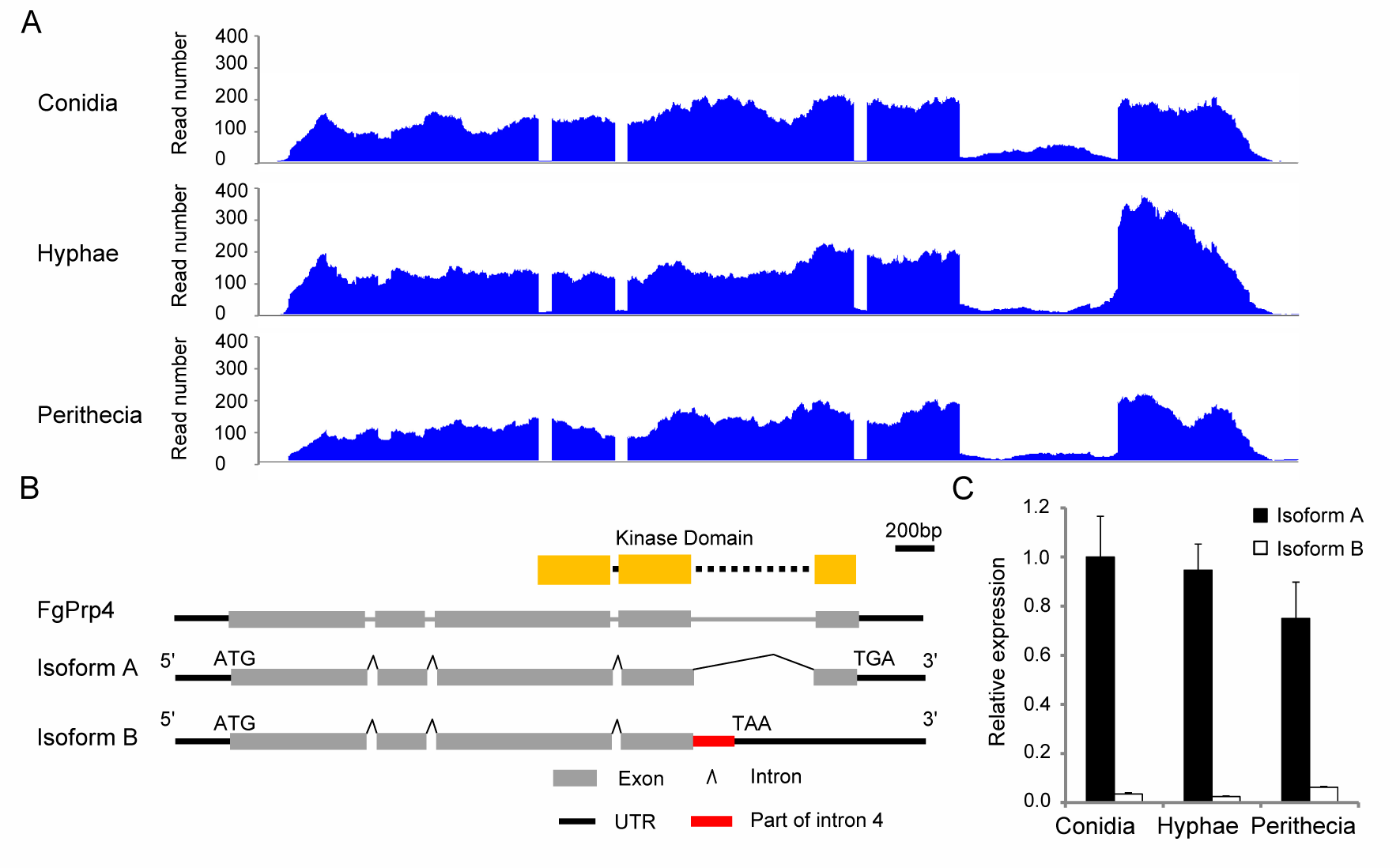

Supplement: S5 Fig — (A). IGV Sashimi plots showing the read numbers and splice junctions of FgPRP4 transcripts in marked RNA-seq data. (B). Schematic draws of FgPRP4 and its two transcript isoforms. The orange boxes are the kinase domain region. (C). qRT-PCR analysis with isoforms A and B of FgPRP4 transcripts. The relative expression level of isoform A in conidia was arbitrarily set to 1. (TIF) [file pgen.1005973.s005.tif]

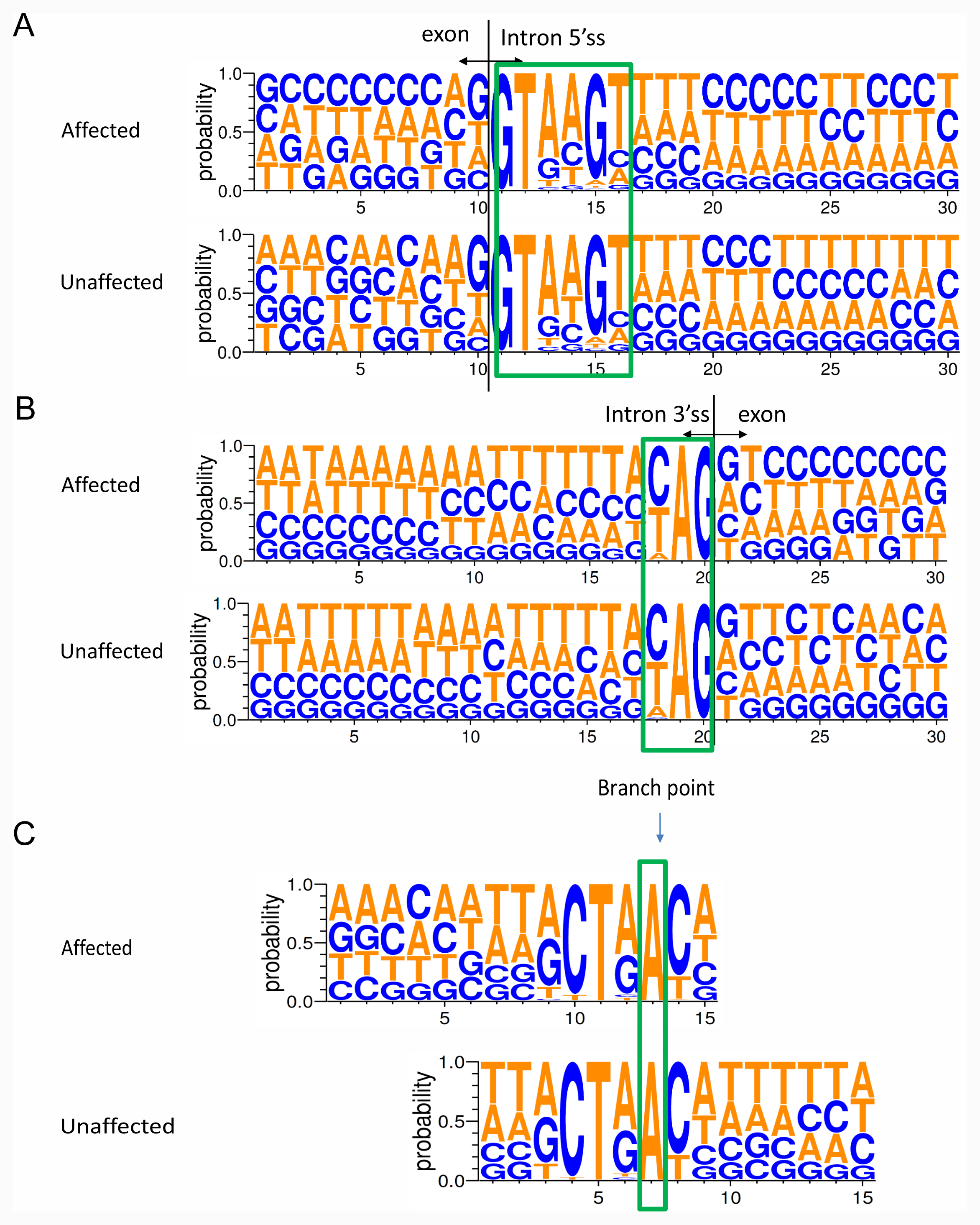

Supplement: S6 Fig — Sequence features of the 5’ss (A), 3’ss (B), and BP (C) of the introns that were significantly affected or not affected by FgPRP4 deletion in splicing efficiency. The 5’ss, 3’ss and BP sequences are marked by green rectangles. (TIF) [file pgen.1005973.s006.tif]

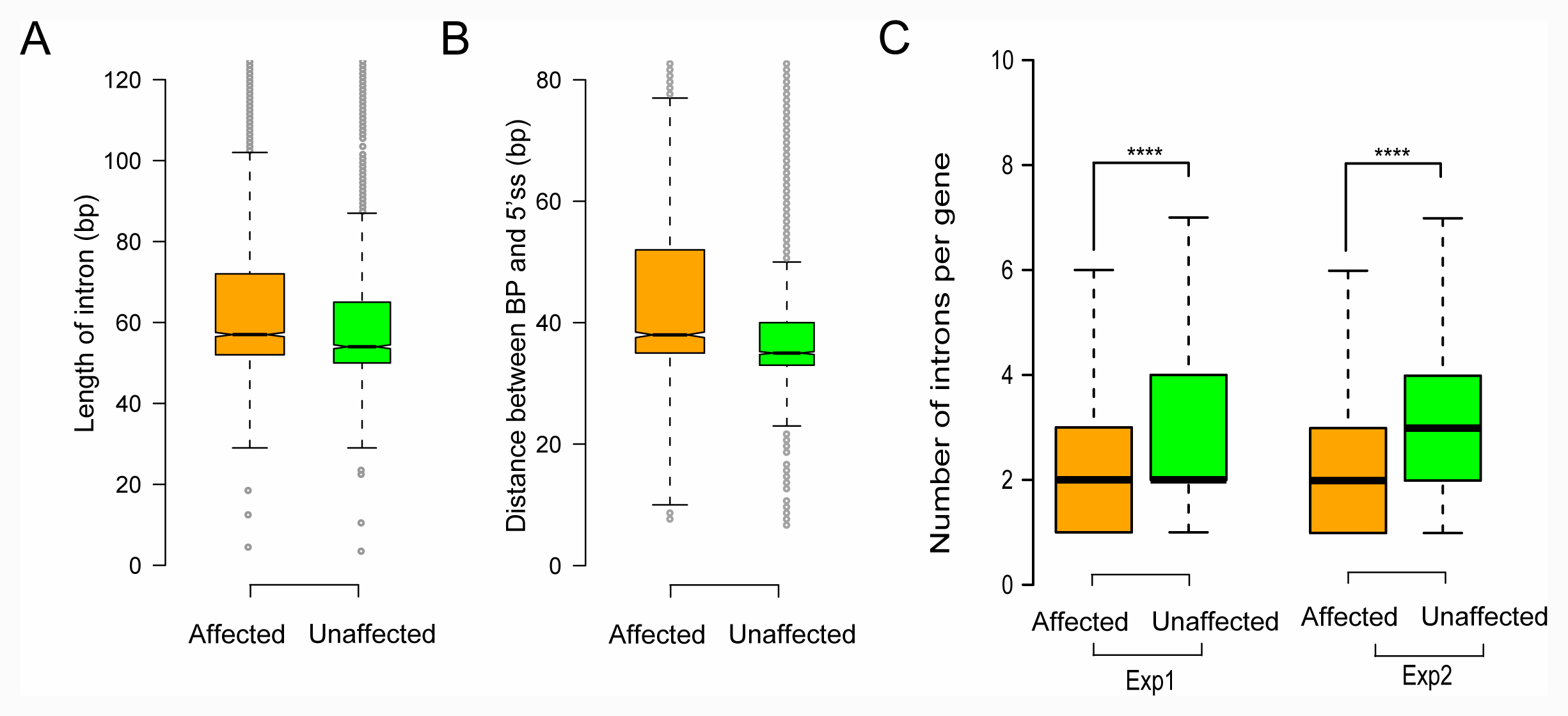

Supplement: S7 Fig — (A). Introns with reduced splicing efficiency in the Fgprp4 mutant tend to be longer than introns unaffected by FgPRP4 deletion (P<0.001). (B). The distance between 5’ss and BP but not the distance between BP and 3’ss is longer in introns affected by FgPRP4 deletion than those not affected. (C). Genes with reduced in intron splicing efficiency in the Fgprp4 mutant tend to have fewer introns than genes not affected by FgPRP4 deletion. ****, P<0.0001. (TIF) [file pgen.1005973.s007.tif]

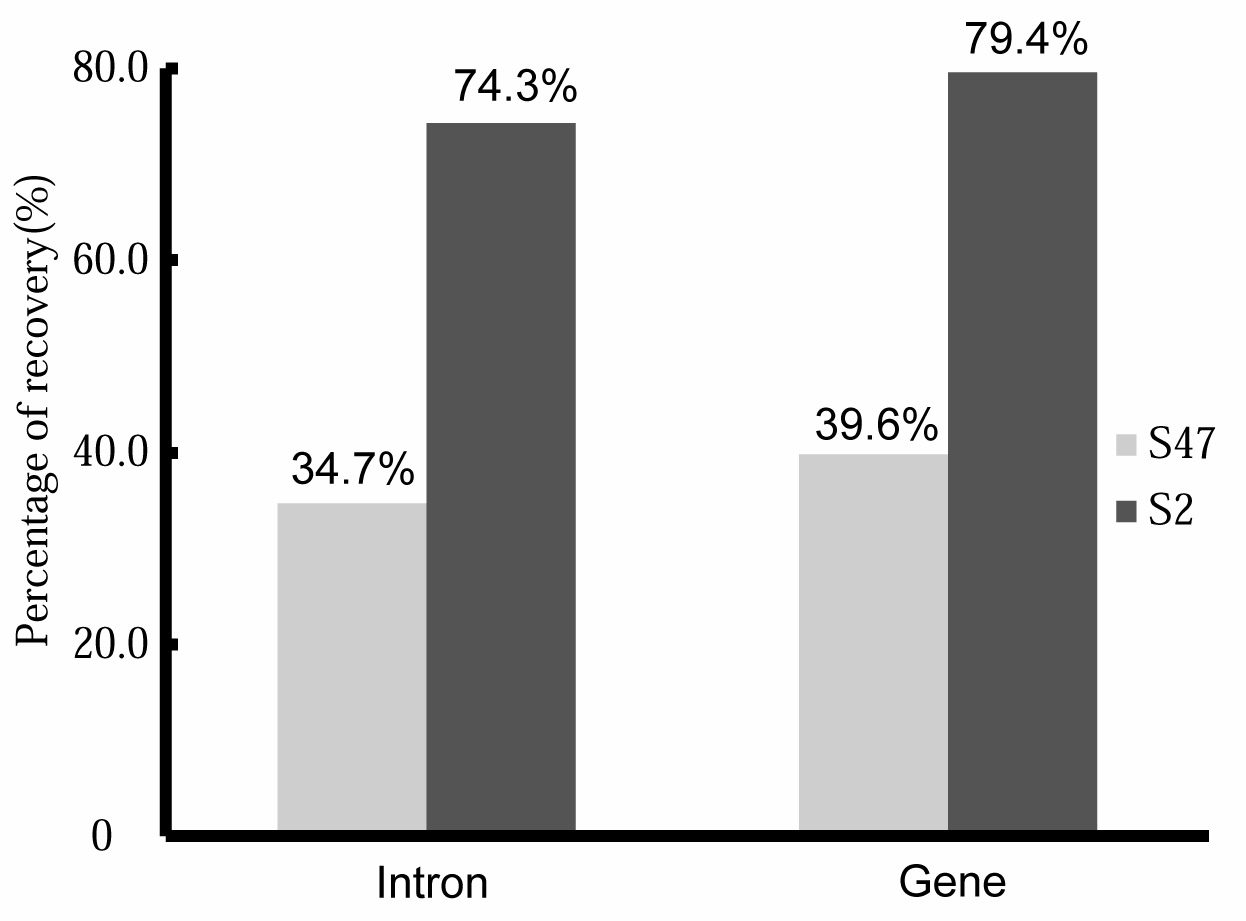

Supplement: S8 Fig — (TIF) [file pgen.1005973.s008.tif]

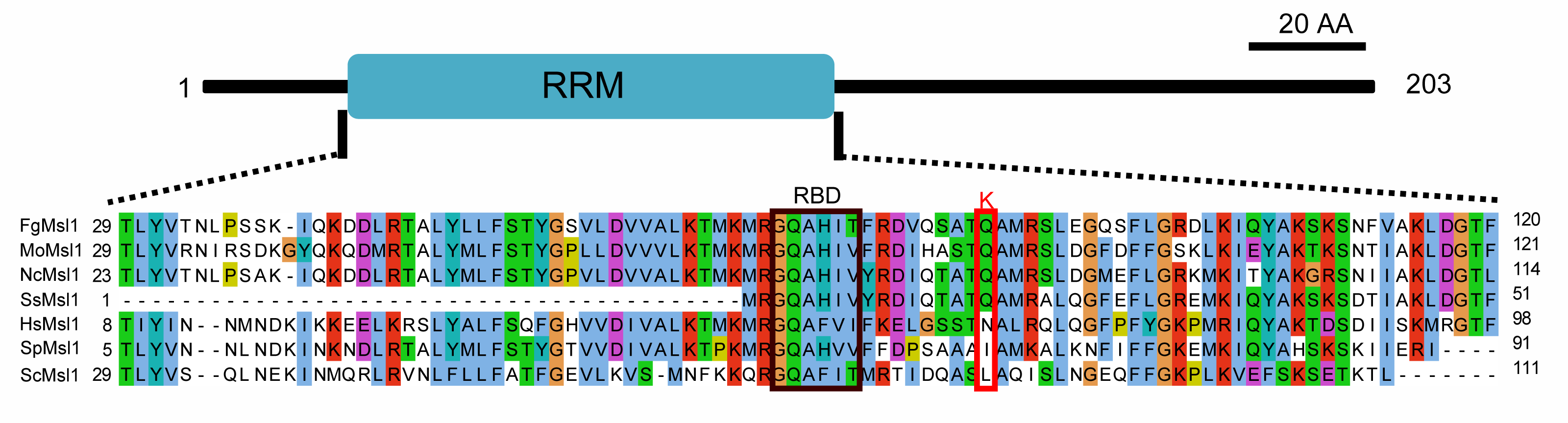

Supplement: S9 Fig — Schematic drawing of the FgMls1 protein structure showed the RRM domain and sequence alignment of its RRM domain of other Mls1 orthologs. The Q86K mutation was labelled on the top and the RBD motif was boxed. (TIF) [file pgen.1005973.s009.tif]

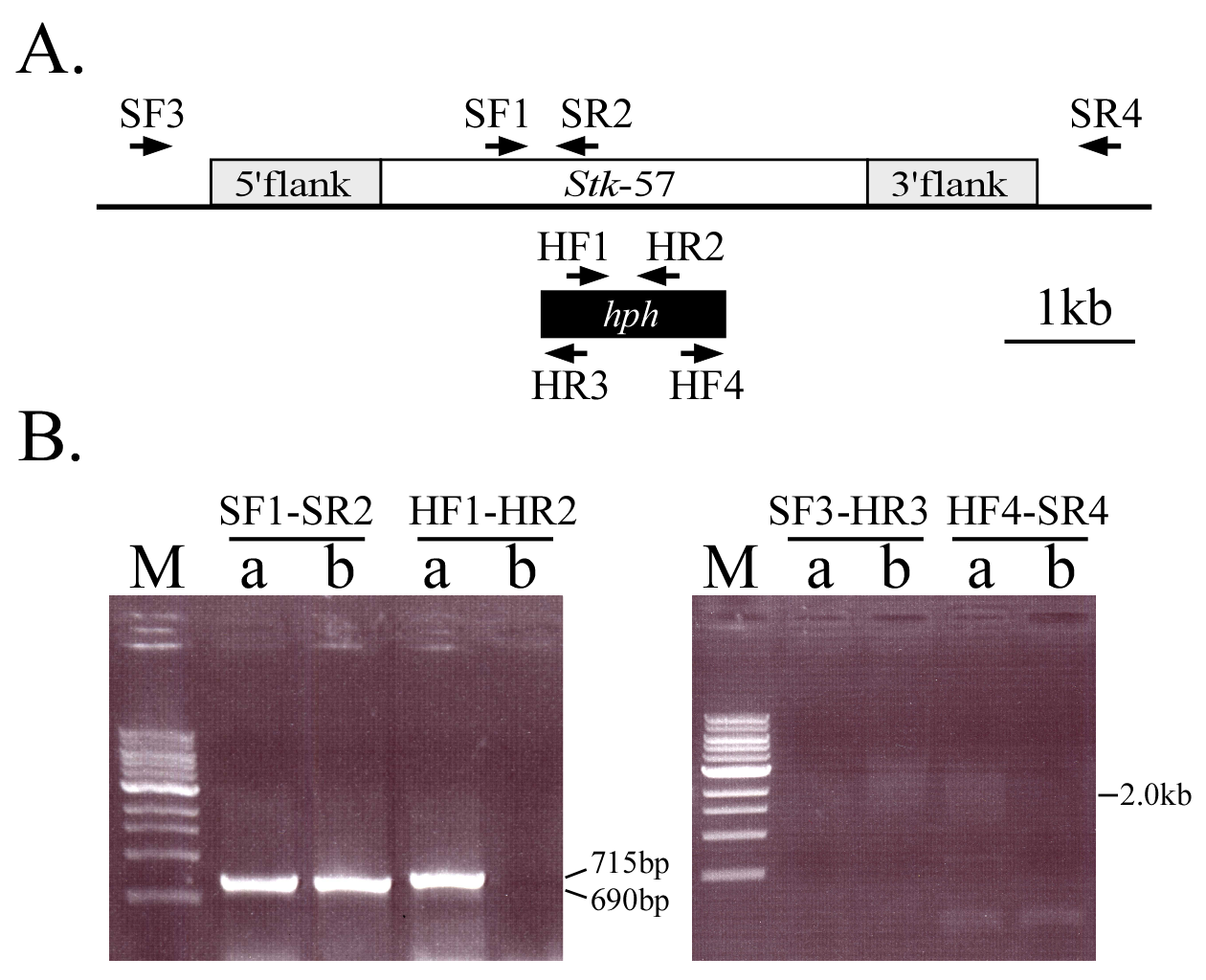

Supplement: S10 Fig — (A) Schematic draw of the STK57 gene, hygromycin-phosphotransferase (hph) cassette, and the positions/directions of PCR primers. (B) PCR analysis with labelled primer pairs with genomic DNA of the wild type (a) and putative stk-57 mutant (b). The expected PCR products amplified by primer pairs SF1/SR2, HF1/HR2, SF3/HR3, and HF4/SR4 were labelled on the side. M, 1 kb DNA Ladder (NEB). (TIF) [file pgen.1005973.s010.tif]
