# Supplementary material for: FgPrp4 Kinase Is Important for Spliceosome B-Complex Activation and Splicing Efficiency in Fusarium graminearum
Source: PLoS Genet. 2016 Apr 8;12(4):e1005973. doi: 10.1371/journal.pgen.1005973 (PMC4825928; doi:10.1371/journal.pgen.1005973)
Supplement: S1 Table — (DOCX) [file pgen.1005973.s011.docx]

**Table S1. Splicing defects in genes related to DNA repair**

|  | **Gene** | **Description** | **Intron** | **Splicing defects** | **% Recovered** | |
| --- | --- | --- | --- | --- | --- | --- |
|  |  |  |  |  | **S2** | **S47** |
| **DNA repair** | *Fg* *SPT16* | SuPpressor of Ty | 1 | 15.39× | 83% | -134% |
|  | *FgPHR1* | PHotoreactivation Repair deficient | 2 | 9.62× | 100% | 100% |
|  | *Fg**POB3* | POl1 Binding | 1 | 7.6× | 88% | 77% |
|  | *Fg**APN1* | APurinic/apyrimidinic eNdonuclease | 2 | 6.9× | 67% | 84% |
|  | *Fg**TFB2* | Transcription initiation Factor IIB | 1 | 5.4× | 47% | -4% |
|  | *FgTEL1* | TELomere maintenance | 9 | 2.5× | 100% | NA |
|  | *FgRAD14* | RADiation sensitive | 1 | 2.8× | 100% | 100% |
|  | *FgSLX1* | Synthetic Lethal of unknown (X) function | 1 | 2.32× | 100% | 100% |
|  | *Fg EAF1* | Esa1p-Associated Factor | 4 | 21.6× | 83% | -26% |
|  | *Fg NHP6A* | Non-Histone Protein | 1 | 15.5× | 68% | 68% |
|  |  |  | 2 | 3.04× | 15% | 66.8% |
