# Supplementary material for: FgPrp4 Kinase Is Important for Spliceosome B-Complex Activation and Splicing Efficiency in Fusarium graminearum
Source: PLoS Genet. 2016 Apr 8;12(4):e1005973. doi: 10.1371/journal.pgen.1005973 (PMC4825928; doi:10.1371/journal.pgen.1005973)
Supplement: S2 Table — (DOC) [file pgen.1005973.s012.doc]

**Table S2. Defects of the suppressor strains in conidiation, pathogenesis, and sexual reproduction**

| **Strain** | **Type** | **Conidiation** | **Virulence** | **Sexual reproduction** |
| --- | --- | --- | --- | --- |
| PH-1 (WT) | Wildtype | Normal | Normal | Normal |
| FP1 (*Fgprp4*) | Mutant | Rare | - **^b^** | Sterile |
| FPC1 | Compleme-ntation | Normal | Normal | Normal |
| S1 | I | Normal | - | Normal |
| S2***^a^** | I | Reduced | Normal | Normal |
| S3* | I | Normal | Normal | Reduced fertility |
| S4 | I | Reduced | Normal | Normal |
| S5* | I | Normal | Reduced | Reduced fertility |
| S6 | II | Normal | - | N/A |
| S7 | I | Reduced | Normal | Normal |
| S8 | II | Normal | - | Normal |
| S9* | I | Normal | - | Normal |
| S10* | I | Reduced | Reduced | Normal |
| S11 | II | Reduced | - | N/A |
| S12 | II | Reduced | - | Sterile |
| S13 | II | Rare | - | Small perithecia, no ascospores |
| S14 | II | Normal | - | Small perithecia, no ascospores |
| S15 | II | Rare | - | No perithecia |
| S16 | I | Normal | - | Rare small perithecia, no ascospores |
| S17* | I | Normal | - | Normal |
| S18 | I | Normal | - | Normal |
| S19* | I | Normal | Reduced | Normal |
| S20 | II | Normal | - | Reduced fertility |
| S21* | I | Normal | Normal | Normal perithecia, no ascospores |
| S22* | I | Reduced | - | Normal |
| S23 | II | Normal | - | Sterile |
| S24 | II | Normal | - | Rare perithecia, no ascospores |
| S25* | I | Normal | Reduced | Normal |
| S26 | I | Normal | Reduced | Normal |
| S27* | I | Normal | Reduced | Normal |
| S28* | I | Normal | Normal | Defective in ascospore development |
| S29 | I | Normal | - | Rare small perithecia, no ascospores |
| S30* | I | Normal | Reduced | Normal |
| S31 | I | Normal | Normal | Normal |
| S32 | I | Normal | Reduced | Sterile |
| S33* | II | Normal | Reduced | Sterile |
| S34* | I | Rare | Reduced | Normal |
| S35 | II | Normal | - | Normal |
| S36* | II | Normal | Reduced | Sterile |
| S37 | I | Reduced | - | Normal |
| S38 | I | Normal | - | Rare perithecia, no ascospores |
| S39* | I | Normal | Reduced | Normal |
| S40 | II | Normal | - | Sterile |
| S41* | II | Reduced | - | Sterile |
| S42 | I | Normal | Normal | Sterile |
| S43* | I | Reduced | - | Normal |
| S44 | I | Rare | - | Small perithecia, no ascospores |
| S45* | II | Normal | - | Sterile |
| S46* | I | Reduced | - | Normal |
| S47* | I | Normal | - | Reduced fertility |
| S48 | I | Rare | - | No perithecia |
| S49 | II | Normal | - | Normal |

**^a^** * marks the suppressor strains selected for sequencing candidate FgPrp4-targets.

**^b^** –non-pathogenic or only caused symptoms on inoculated kernels.

N/A, not assayed.

**Table S6. Candidate Prp4-target genes sequenced in the selected suppressor strains**

|  | **Fg10242** | **Fg01210** | **Fg01299** | **Fg02536** | **Fg01337** | **Fg01024** | **Fg09864** | **Fg04292** | **Fg10001** | **Fg06745** |
| --- | --- | --- | --- | --- | --- | --- | --- | --- | --- | --- |
| Sc | *PRP6* | *BRR2* | *PRP31* | *PRP8* | *PRP46* | *PRP5* | N/A | *PRP3* | *SNU13* |  |
| Sp | *PRP1* | *SPP41*  *(brr2)* | *PRP31* | *SPP42/* (*CSF6*) | *PRP5*  (*CWF1*) | *PRP11* | *SRP1* | *PRP3* | *SNU13* (*PRP6*) | *PNN1* |
| Human | *102K, PRPF6* | *200K, SNRNP200* | *61K, PRPF31* | *220K, PRPF8* | *102K, PLRG1* | *DDX46* | *SRFS2* | *90K, PRPF3* | *15.5K, NHP2L1* | *pinin, PNN* |
| S2 | - | - | R464* | - | - | - | - | - | NS | NS |
| S3 | - | - | - | - | - | - | - | - | - | - |
| S5 | - | - | - | - | - | - | - | - | - | - |
| S9 | - | - | - | - | - | - | - | - | - | - |
| S10 | - | - | - | - | - | - | - | - | - | - |
| S17 | - | - | L532P | - | - | - | - | - | NS | NS |
| S19 | - | - | - | - | - | - | - | - | - | - |
| S21 | - | - | - | - | - | - | - | - | - | - |
| S22 | R230C | - | - | - | - | - | - | - | NS | NS |
| S25 | - | - | - | - | - | - | - | - | - | - |
| S27 | - | - | - | - | - | - | - | - | - | - |
| S28 | - | - | - | - | - | - | - | - | - | - |
| S30 | - | G308E | - | - | - | - | - | - | NS | NS |
| S33 | - | - | - | - | - | - | - | - | - | - |
| S34 | - | - | - | D1153G | - | - | - | - | NS | NS |
| S36 | - | - | - | - | - | - | - | - | - | - |
| S39 | ΔE309 | - | - | - | - | - | - | - | NS | NS |
| S41 | - | - | - | - | - | - | - | - | - | - |
| S43 | - | - | - | E1429K | - | - | - | - | NS | NS |
| S45 | - | - | - | - | - | - | - | - | - | - |
| S46 | ΔE309 | - | - | - | - | - | - | - | NS | NS |
| S47 | R230H | - | - | - | - | - | - | - | NS | NS |

-, sequenced but no changes found

NS, not sequenced

*, stop codon
