# Supplementary material for: FgPrp4 Kinase Is Important for Spliceosome B-Complex Activation and Splicing Efficiency in Fusarium graminearum
Source: PLoS Genet. 2016 Apr 8;12(4):e1005973. doi: 10.1371/journal.pgen.1005973 (PMC4825928; doi:10.1371/journal.pgen.1005973)
Supplement: S3 Table — (DOC) [file pgen.1005973.s013.doc]

**Table S3. Primers used in the study.**

| Name | | Sequences (5’ – 3’) |
| --- | --- | --- |
| 04053/1F | CAACAGCATGGATCGTCGGG | |
| 04053/2R | TTGACCTCCACTAGCTCCAGCCAAGCCCAGGTCGTGATGTCGCAAACTC | |
| 04053/3F | GAATAGAGTAGATGCCGACCGCGGGTTTGTCACCGCCGTTCGTATGT | |
| 04053/4R | CCCGAGTAATGCCCTTCACC | |
| 04053/5F | CCATCGCAACAGAACCACAGC | |
| 04053/6R | CCCCAGCCACCATAGTAAGAATAGT | |
| 04053/7F | GCCTTTAGAAGTGTCGCAGAA | |
| 04053/8R | CTTTCTGACTTGGTATCCTTGACT | |
| H850 | TTCCTCCCTTTATTTCAGATTCAA | |
| H852 | ATGTTGGCGACCTCGTATTGG | |
| H855R | GCTGATCTGACCAGTTGC | |
| H856F | GTCGATGCGACGCAATCGT | |
| HY/R | GTATTGACCGATTCCTTGCGGTCCGAA | |
| YG/F | GATGTAGGAGGGCGTGGATATGTCCT | |
| HYG/F | GGCTTGGCTGGAGCTAGTGGAGGTCAA | |
| HYG/R | AACCCGCGGTCGGCATCTACTCTATTC | |
| 04053HB/F | CGGGGTACCCCTTTAGAAGTGTCGCAGAA | |
| 04053HB/R | CGCGGATCCCGTAGGCATTTGTTCCAG | |
| 04053gfp/F | CGACTCACTATAGGGCGAATTGGGTACTCAAATTGGATGGAAAGACACGCACCGAGGC | |
| 04053gfp/R | CACCACCCCGGTGAACAGCTCCTCGCCCTTGCTCACGCGACGTATAGGCCCCGCCTTC | |
| 04053flag/R | CTTTATAATCACCGTCATGGTCTTTGTAGTCGCGACGTATAGGCCCCGCCT | |
| 04053-289S-f | TGCTGTAGGAGCTGCAGCACGCGCTGCAGCACCTGCTTCCACCCTCCCCGACACG | |
| 04053-289S-r | CGTGTCGGGGAGGGTGGAAGCAGGGGCTGCAGCGCGTGCTGCAGCTCCTACAGCA | |
| 04053-310d-f | CCAATAAGTACAGTCGTCAAAATGCCTCGATCAGACATAGCATCACC | |
| 04053-310d-r | GGTGATGCTATGTCTGATCGAGGCATTTTGACGACTGTACTTATTGG | |
| 04053dl/F | ACTACCATCAAAACCTTGGCTGTTG | |
| 04053dl/R | CAACGCAAAGAAGGGATGGC | |
| 04053dl-ie/F | GGGACAGTAACAACCAGATGCT | |
| 04053dl-in/R | CCCAGCCACCATAGTAAGAATA | |
| 04053dl-ex/R | GATGGTAGTCTTGTCCAAAACCTTG | |
| 10242gfp/F | CGACTCACTATAGGGCGAATTGGGTACTCAAATTGACCGTCAAAGCACATAACCCT | |
| 10242gfp/R | CACCACCCCGGTGAACAGCTCCTCGCCCTTGCTCACTTGTTCCAGCTCTTCAGCAACCAG | |
| 01299sq/1F | AGGTCGTCGCCATCATAGGAA | |
| 01299sq/2R | CAGATACGGCAGAGAAATCGCAACCTCTCAGGTACGCTGGTCGATCTGAGTA | |
| 01299sq/3F | GTTTAGATTCCAAGTGTCTACTGCTGGCTCAAAACAGAACATCATCCTC | |
| 01299sq/4R | CAACACAGCGAACCTGCCAAG | |
| 01299sq/7F | GAGGTCGCTCTCGTCCATAAA | |
| 01299sq/8R | TCTGGCGGTGTAAAGGGTCTCT | |
| 01299sq/CF | CAGGCGGGCTCACAGGTCT | |
| 01299sq/CR | GTGATGTGGCAGGGCATAAGA | |
| GEN/F | GAGGTTGCGATTTCTCTGCCGTATCTG | |
| GE/R | CCACAGTCGATGAATCCAGAAAAGCG | |
| EN/F | GGAAGGGACTGGCTGCTATTGG | |
| GEN/R | GCCAGCAGTAGACACTTGGAATCTAAAC | |
| G852/F | TCGGCTATGACTGGGCACAACA | |
| G850/R | GAGCGGCGATACCGTAAAGCAC | |
| G855/R | TGTTGGGTTTGAGCTAGGTGGG | |
| G856/F | GAATGGTCAAATCAAACTGCTAGATAT | |
| 01299sqs17/1F | GAAGTTGACAGAGCCACCGCC | |
| 01299sqs17/2R | CAGATACGGCAGAGAAATCGCAACCTCTCATTTCGATGCTGAGGGACCTG | |
| 01299sqs17/3F | GTTTAGATTCCAAGTGTCTACTGCTGGCTCAAAACAGAACATCATCCTC | |
| 01299sqs17/4R | CAACACAGCGAACCTGCCAAG | |
| 01299sqs17/7F | ATTCCAAGTGACCTGAGAAAGCA | |
| 01299sqs17/8R | TCTGGCGGTGTAAAGGGTCTCT | |
| 01299sqs17/CF | TGACGGACCTTCGCAAACAAC | |
| 01299sqs17/CR | GTGATGTGGCAGGGCATAAGA | |
| cx01299/1F | GACTCCATCTTGTGCTATTCGCTTT | |
| cx01299/2R | CCGACAAGAGCGGTCAAAT | |
| cx01299/3F | CTCTCAAGTCTGTCCTGGATGGG | |
| cx01299/4R | CACCCTCCATGTTTCCACAGTACA | |
| cx10242/1F | CGGAGCAAGGTTAGGGATGGA | |
| cx10242/2R | CCCAGAGTTTCACGGATAATGT | |
| cx10242/3F | CAACGAGGGCTCACCCAATG | |
| cx10242/4R | TCAAGGTCGGAGCCTACGGTTA | |
| cx01210/1F | CTCCATACAATCTTTCAACGCCTG | |
| cx01210/2R | ATTTGTTGCTTGGTGAGTTGGCGA | |
| cx01210/3F | GTCCAGGAGCAGGTGGGCAAT | |
| cx01210/4R | TTCGGCGTATTCCTTGCGTAAC | |
| cx01210/5F | CCAAGTTCCCCCGTGTTGAGGT | |
| cx01210/6R | GCCCAACGAAAGGATGAAGGTCTG | |
| cx01210/7F | TCAACTGGACCACCTTTACCTATT | |
| cx01210/8R | TCAAAGAGCACATCTCAGTCACAC | |
| cx02536/1F | Cagacaggtcgtaaatacagggat | |
| cx02536/2R | Atcaggatcgtctgctcggttg | |
| cx02536/3F | Tccctcagatggcgactctattc | |
| cx02536/4R | Gattgaggcgtccctttactga | |
| cx02536/5F | Ggtgggtttcagtcgctcat | |
| cx02536/6R | Gcgattctgttggttggcttc | |
| cx02536/7F | Tgccttctcgtttccctcct | |
| cx02536/8R | Atggcaccgttgatgggcttg | |
| cx02536/9F | Ccaaccctgctctgtatgtcct | |
| cx02536/10R | Cgatggttcttcggctctgc | |
| cx04292/1F | GCGTTGCCATCTACCTATTTGC | |
| cx04292/2R | CAGACGACGGAGCCCAGTGTT | |
| cx00884/1F | CGTCGCTCTTCCGATTTAGG | |
| cx00884/2R | CTTTGGTCTCGTATCTCCCTCC | |
| cx13556/1F | CACGGCTGTTGCGAATACG | |
| cx13556/2R | TCTCCAACAACCTCTCGGCAG | |
| cx13556/3F | TGCGAGCAGTTCTTTGGACCT | |
| cx13556/4R | CGTTTGTTCCTGATGGCGGTC | |
| cx06849/1F | AAGCCTGACCAATGAAGAGCAAG | |
| cx06849/2R | CGAGAGACAGATGAGAAGAGGCT | |
| cx02648/1F | AACATCGCCCTGGGAAGTCG | |
| cx02648/2R | CTTTTCTAAGCCGTCGCCGC | |
| cx06996/1F | CCATCGTCCATAACAAACTACCA | |
| cx06996/2R | GTAAGGCTTCACTACACGGACTC | |
| 00385rt/1F | AAGCGTGCGACCAGAACTAA | |
| 00385rt/2R | GACCGAAAGAGATACCAGGG | |
| 00797rt/1F | ATCGTCGCTTCATTTCTGGC | |
| 00797rt/2R | AGGAACCCACTTGCGGATGT | |
| 05512rt/1F | CACCCAGTCTTGACCAACCCA | |
| 05512rt/2R | GTCGGCGGAACGAGTAACA | |
| SF1 | TTCTGCGTTCAACTTCTTCC | |
| SR2 | CGGAGGTTCATGCTCAAATT | |
| SF3 | CCCAGGAAGACGTCAAAAGG | |
| SR4 | GTGAAGCGAGCAAGTGAAGT | |
| HF1 | ACGTCTGTCGAGAAGTTTCT | |
| HR2 | GCGTCTGCTGCTCCATACAA | |
| HR3 | CTACTGCTTACAAGTGGGCTGA | |
| HF4 | TCTGGACCGATGGCTGTGTAG | |
